# Trait adaptation enhances species coexistence and reduces bistability in an intraguild predation module

**DOI:** 10.1002/ece3.9749

**Published:** 2023-01-23

**Authors:** Xiaoxiao Li, Toni Klauschies, Wei Yang, Zhifeng Yang, Ursula Gaedke

**Affiliations:** ^1^ Guangdong Provincial Key Laboratory of Water Quality Improvement and Ecological Restoration for Watersheds, School of Ecology, Environment and Resources Guangdong University of Technology Guangzhou China; ^2^ State Key Laboratory of Water Environment Simulation, School of Environment Beijing Normal University Beijing China; ^3^ Southern Marine Science and Engineering Guangdong Laboratory (Guangzhou) Guangzhou China; ^4^ Department of Ecology and Ecosystem Modelling Institute of Biochemistry and Biology, University of Potsdam Potsdam Germany; ^5^ Yellow River Estuary Wetland Ecosystem Observation and Research Station Ministry of Education Shandong China

**Keywords:** alternative stable states, eco‐evolutionary dynamics, enrichment, intraspecific trait variation, predator–prey dynamics, rapid evolution, trade‐offs

## Abstract

Disentangling how species coexist in an intraguild predation (IGP) module is a great step toward understanding biodiversity conservation in complex natural food webs. Trait variation enabling individual species to adjust to ambient conditions may facilitate coexistence. However, it is still unclear how coadaptation of all species within the IGP module, constrained by complex trophic interactions and trade‐offs among species‐specific traits, interactively affects species coexistence and population dynamics. We developed an adaptive IGP model allowing prey and predator species to mutually adjust their species‐specific defensive and offensive strategies to each other. We investigated species persistence, the temporal variation of population dynamics, and the occurrence of bistability in IGP models without and with trait adaptation along a gradient of enrichment represented by carrying capacity of the basal prey for different widths and speeds of trait adaptation within each species. Results showed that trait adaptation within multiple species greatly enhanced the coexistence of all three species in the module. A larger width of trait adaptation facilitated species coexistence independent of the speed of trait adaptation at lower enrichment levels, while a sufficiently large and fast trait adaptation promoted species coexistence at higher enrichment levels. Within the oscillating regime, increasing the speed of trait adaptation reduced the temporal variability of biomasses of all species. Finally, species coadaptation strongly reduced the presence of bistability and promoted the attractor with all three species coexisting. These findings resolve the contradiction between the widespread occurrence of IGP in nature and the theoretical predictions that IGP should only occur under restricted conditions and lead to unstable population dynamics, which broadens the mechanisms presumably underlying the maintenance of IGP modules in nature. Generally, this study demonstrates a decisive role of mutual adaptation among complex trophic interactions, for enhancing interspecific diversity and stabilizing food web dynamics, arising, for example, from intraspecific diversity.

## Graphical abstract

We found that trait adaptation within multiple species greatly enhanced the coexistence of all species in the module. Increasing the speed of trait adaptation buffered the detrimental effects of enrichment on the temporal variability of biomasses of all species. The bistability encountered in the non‐adaptive IGP model was strongly reduced when allowing trait adaptation.

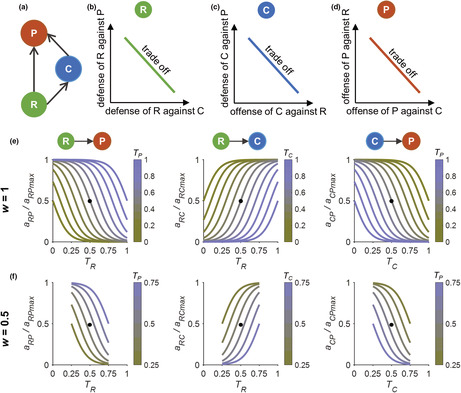

## 1 INTRODUCTION

The intraguild predation (IGP) module, comprising an omnivore (IG predator) competing with its prey (IG prey) for a shared basal prey, is recognized as a ubiquitous feature in natural food webs (Arim & Marquet, 2004; Kratina et al., 2012; Novak, 2013). This strongly contrasts with early predictions of IGP models, suggesting that IGP modules should be rare in nature due to their destabilizing effect on species' population dynamics (Pimm & Lawton, 1978). In addition, coexistence of all three species was only possible for intermediate levels of enrichment represented by the carrying capacity of the basal prey, and relied on the assumptions that the IG prey must be the superior competitor in exploiting the basal prey, while the IG predator must gain greatly from consuming the IG prey (Diehl & Feißel, 2000; Holt & Polis, 1997). Such models also predicted that IGP modules tend to be very fragile due to the existence of alternative stable states with different species persisting, which further affects the stability of communities and their resilience to external perturbations (Holt & Polis, 1997; Mylius et al., 2001; Verdy & Amarasekare, 2010).

Such discrepancies raise the question, which additional ecological or evolutionary processes need to be incorporated into IGP models to obtain a better match between empirical findings and theoretical predictions. Most previous theoretical studies assumed that the functional traits of the component species were constant, despite increasing evidence that organisms can adapt to altered environmental conditions on ecologically relevant timescales (Abrams, 2000; Fussmann et al., 2007; Hairston et al., 2005; van Velzen & Gaedke, 2018). In nature, trophic interactions often dynamically vary due to phenotypic plasticity or rapid evolution of species, such as switching foraging of the consumer or changing defense of the prey. Such potentials for trait adaptation have far‐reaching consequences for species persistence, population dynamics, and alternative states of entire communities (Schoener, 2011; van Velzen & Gaedke, 2018; Yoshida et al., 2003).

Hence, recent studies explored the potentially important role of trait adaptation within IGP modules by allowing individual species to adjust their traits in response to selection. Some studies assumed a universal antipredator defense of the basal prey that protected it against predation by both predators at the cost of a reduced growth rate (Ikegawa et al., 2015; Kimbrell et al., 2007), whereas others considered trait variation within the basal prey that mediated predator‐specific defenses (Ellner & Becks, 2011; Hiltunen et al., 2013; Nakazawa et al., 2010). Trait variation within the IG prey was subject to a trade‐off between an effective antipredator defense and a high feeding rate on the basal prey (Urbani & Ramos‐Jiliberto, 2010; Visser et al., 2012). Finally, considering trait variation within the IG predator usually included a trade‐off between efficiently feeding on the basal prey or the IG prey (Křivan & Diehl, 2005; Michalko & Pekár, 2017; Patel & Schreiber, 2015). Some studies also analyzed the impact of coadaptation by jointly incorporating trait variation within two different species, for example, within the IG prey and the IG predator (Fung, 2008) or the basal prey and the IG predator (Ikegawa et al., 2015). Allowing for trait adaptation within one or two different species generally enhanced the coexistence of all three species and mostly stabilized population dynamics.

However, since, in nature, all species exhibit at least some potential to adjust their trait values to ambient environmental conditions, it is more realistic to assume that all species of an IGP model mutually adapt to each other (Gravel et al., 2016; Valdovinos et al., 2010). Analyzing this scenario is particularly relevant as it is impossible to predict the population dynamics of such a fully adaptive IGP model from models that only allow individual species to adapt their trait values in response to selection as potentially important trait‐mediated indirect effects on trophic interactions are neglected that may only arise when all species are adaptive (Abdala‐Roberts et al., 2019; Vance‐Chalcraft et al., 2007). Indeed, trait adaptation of different species may strongly interact with each other and jointly affect the shape of population dynamics and relevant ecosystem functions (Levine et al., 2017; Schmitz et al., 2004). For example, when the IG prey adapts its traits to exploit the basal prey more efficiently, the basal prey may respond to defend more against the predation by the IG prey. Meanwhile, the IG predator will adapt its species‐specific offense traits to maximize its consumption depending on the defense of both prey. Tirok et al. (2011) found that more complex community dynamics, for example, intermittent cycles, occurred when prey and predator could coadapt but not when only a single species was adaptive. Therefore, it is still open how species coexistence, population dynamics, and the occurrence of alternative states in an IGP model are affected if all species are adaptive.

**FIGURE 1:**
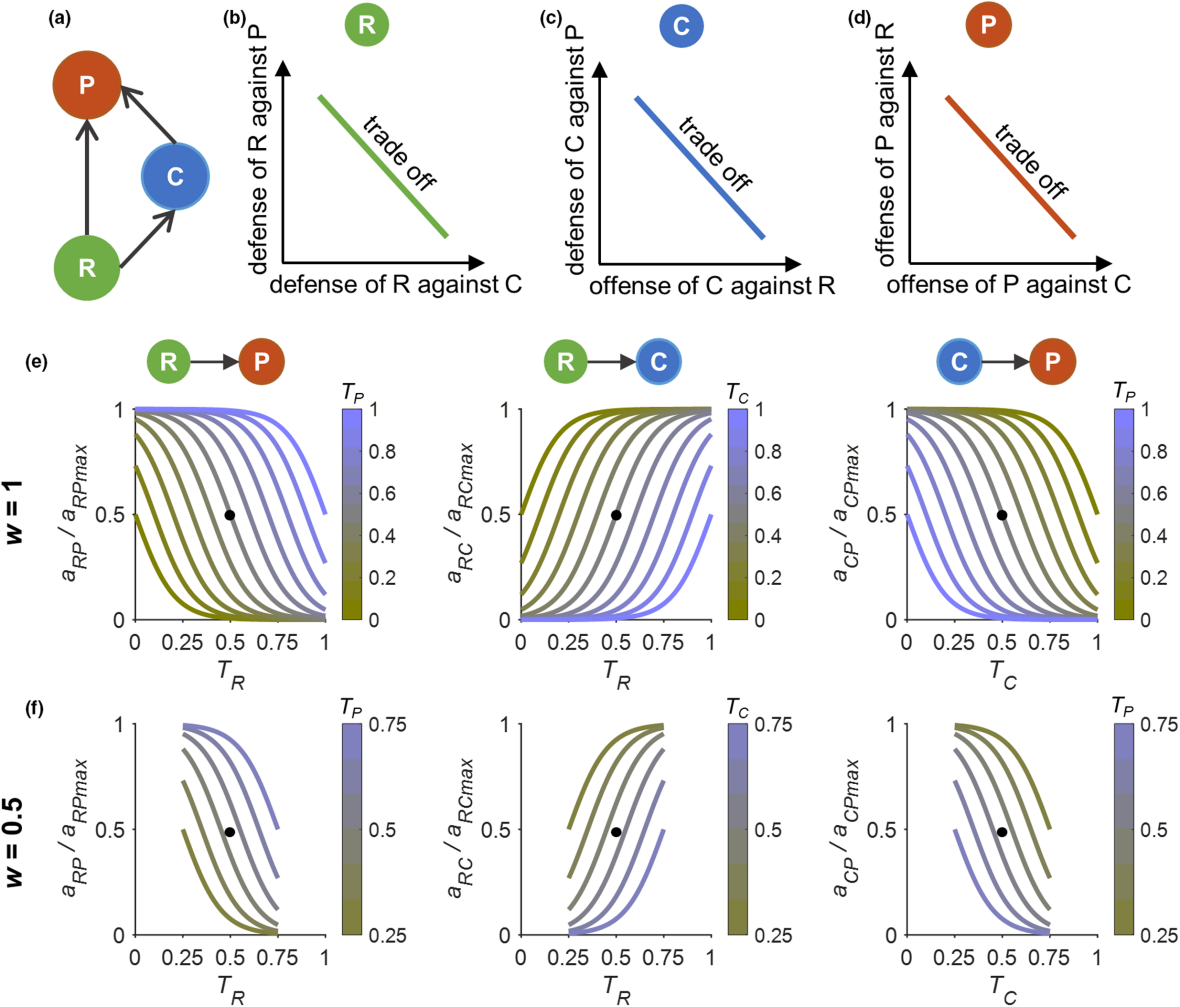
(a) Structure of an intraguild predation (IGP) module consisting of the basal prey (R), the IG prey (C), and the IG predator (P). (b–d) The trade‐offs between species‐specific trait adaptations of the three species are: for R, its defense against C trades off with its defense against P; for C, its offense trait against the defense of R trades off with its defense trait against P; for P, its offense trait against the defense of C trades off with its offense against the defense of R. (e,f) Standardized attack rate of P on R (*a*_*RP*_), standardized attack rate of C on R (*a*_*RC*_), and standardized attack rate of P on C (*a*_*CP*_) as functions of species trait values (*T*_*i*_) under different assumed width (*w*) of trait adaptation. For the maximum attack rate values, see Table 1. Black dots indicate the fixed trait values and attack rates in nonadaptive IGP models

Our study tackles this issue by developing an adaptive IGP model (Figure 1a) that allows (1) predator‐specific defense adaptation of the basal prey (Figure 1b), that is, the defense of the basal prey against the IG prey is assumed to trade‐off with its defense against the IG predator; (2) foraging‐defense adaptation of the IG prey (Figure 1c), that is, the offense of the IG prey against the basal prey (that is the capacity to counteract the defense of the basal prey) trades off with its defense against the IG predator; and (3) prey‐specific foraging adaptation of the IG predator, that is, the offense of the IG predator against defense of the IG prey trades off with its offense against defense of the basal prey (Figure 1d). We considered the effects of both the width and the speed of trait adaptation (cf. Klauschies et al., 2016). We first compared species persistence and biomass dynamics in IGP models without and with trait adaptation for the same ecologically reasonable parameter space defined by enrichment (represented by the carrying capacity of the basal prey) and the maximum attack rate of the IG predator on the basal prey, which were decisive parameters in nonadaptive IGP models (Holt & Polis, 1997; Mylius et al., 2001). Then, we investigated the combined effects of the width and speed of trait adaptation on the dynamics of our adaptive IGP model along a gradient of enrichment. Finally, we compared the occurrence of alternative states (i.e., bistability), which has been established for the nonadaptive model (Holt & Polis, 1997; Mylius et al., 2001), between the two types of model for a large parameter space.

## 2 MATERIALS AND METHODS

Based on a classical intraguild predation (IGP) model (Holt & Polis, 1997) and recent adaptive trait analyses (Abrams, 2010; Klauschies et al., 2016), we developed an adaptive IGP model, which tracks the dynamics of the biomasses and traits of the three different species, that is, the basal prey (R), the IG prey (C), and the IG predator (P). Our adaptive IGP model incorporated species‐specific traits that mediate the predator‐specific defense of the prey and the prey‐specific offense of the predators, allowing us to examine how trait dynamics of all three species jointly affect species persistence and biomass dynamics in the IGP module.

### 2.1 Dynamics of species biomasses

The biomass dynamics of the three species are described as follows:

(1) $$\frac{\mathrm{dR}}{\mathrm{dt}}=\mathrm{Rr}\left(1-\frac{R}{K}\right)-{ℊ}_{\mathrm{RC}}-{ℊ}_{\mathrm{RP}}$$

(2) $$\frac{\mathrm{dC}}{\mathrm{dt}}=e{ℊ}_{\mathrm{RC}}-{ℊ}_{\mathrm{CP}}-d_{C}C$$

(3) $$\frac{\mathrm{dP}}{\mathrm{dt}}=e{ℊ}_{\mathrm{RP}}+{eℊ}_{\mathrm{CP}}-d_{P}P$$

where *r* and *K* denote the intrinsic growth rate and carrying capacity of R, *e* the conversion efficiency, and *d*_*C*_ and *d*_*P*_ the mortality rates of C and P, respectively. The prey–predator interactions are described by a function ℊ that is modeled as a multispecies Holling type II functional response:

(4) $${ℊ}_{\mathrm{RP}}=\frac{a_{\mathrm{RP}}\left(T_{R},T_{P}\right)\mathrm{RP}}{1+h_{\mathrm{RP}}a_{\mathrm{RP}}\left(T_{R},T_{P}\right)R+h_{\mathrm{CP}}a_{\mathrm{CP}}\left(T_{C},T_{P}\right)C}$$

(5) $${ℊ}_{\mathrm{RC}}=\frac{a_{\mathrm{RC}}\left(T_{R},T_{C}\right)\mathrm{RC}}{1+h_{\mathrm{RC}}a_{\mathrm{RC}}\left(T_{R},T_{C}\right)R}$$

(6) $${ℊ}_{\mathrm{CP}}=\frac{a_{\mathrm{CP}}\left(T_{C},T_{P}\right)\mathrm{CP}}{1+h_{\mathrm{RP}}a_{\mathrm{RP}}\left(T_{R},T_{P}\right)R+h_{\mathrm{CP}}a_{\mathrm{CP}}\left(T_{C},T_{P}\right)C}$$

where *a*_*ij*_ and *h*_*ij*_ denote the attack rate and handling time of the predatory species *j* on its prey species *i,* depending on the trait values *T* of the relevant species. In line with previous studies (Diehl & Feißel, 2000; Urbani & Ramos‐Jiliberto, 2010), we assumed the ability of P to feed on both R and C to come with a cost of being the inferior competitor with respect to R. Hence, P has a lower attack rate and a longer handling time when feeding on R than C (cf. Table 1). In standard model runs, we assumed the same handling time of P feeding on R and C (cf. Table 1). A sensitivity analysis (Figure S1 in the Supporting Information) showed that different handling times of P feeding on R or C had little impact on species coexistence and biomass dominance between C and P in our adaptive IGP model (Figure S1). By contrast, in the nonadaptive model, the parameter space where all three species coexisted was reduced if the handling time of P feeding on C was relatively high.

**TABLE 1:** Standard parameter values and their interpretations (for further specific parametrizations see Figure captions)

Parameter	Interpretation	Value	
		Nonadaptive model	Adaptive model
*K*	Carrying capacity	0.001–20	
*r*_*max*_	Maximum growth rate of the basal prey	1	
*e*	Conversion efficiency	0.3	
*h*_*RC*_	Handling time of the IG prey feeding on the basal prey	0.2	
*h*_*RP*_	Handling time of the IG predator feeding on the basal prey	0.5	
*h*_*CP*_	Handling time of the IG predator feeding on the IG prey	0.5	
*d*_*C*_	Death rate of the IG prey	0.1	
*d*_*P*_	Death rate of the IG predator	0.2	
*a*_*RPmax*_	Maximum attack rate of the IG predator on the basal prey	0.001–1	
*a*_*RCmax*_	Maximum attack rate of the IG prey on the basal prey	1	
*a*_*CPmax*_	Maximum attack rate of the IG predator on the IG prey	1.2	
*σ*	Steepness of the functions of attack rates	–	0.1
*s*	Steepness of the trait boundary functions	–	10
*v*	Speed of trait adaptation	0	0.001–0.1
*w*	Width of trait adaptation	–	0.01–1

### 2.2 Trade‐offs in attack rates

In the nonadaptive IGP model, trait values of all species were kept at 0.5 to obtain intermediate values for the attack rates (cf. Figure 1e,f). In our adaptive IGP model, we allowed species to adapt their traits and therefore to adjust the attack rates according to species‐specific trade‐offs among their different defense and/or offense strategies. Specifically, compared with the constant attack rates in the nonadaptive IGP model, trait values of R (*T*_*R*_) below and above 0.5 indicate a stronger defense against C and a stronger defense against P, respectively; trait values of C (*T*_*C*_) below and above 0.5 refer to a more pronounced offense against R and a stronger defense against P, respectively; and trait values of P (*T*_*P*_) below and above 0.5 represent a more pronounced offense against C and against R, respectively. Such trade‐offs between species‐specific trait adaptations have been frequently observed in nature, such as different predator‐specific morphological adaptations of larval tadpoles of the genus Rana when exposed to predation pressure (Kishida & Nishimura, 2005; Van Buskirk & McCollum, 1999), size‐dependent trade‐offs between foraging gain and predation risk (e.g., for Arctic charr, *Rana temporaria*) (Eklöv & Halvarsson, 2000; L'Abee‐Lund et al., 1993), and alternative foraging modes dependent on the relative prey density (e.g., for juvenile lumpfish) (Killen et al., 2007; Visser & Fiksen, 2013).

In general, the attack rate of a predator on its prey is assumed to increase for higher values of the predator's offense trait and lower values of the prey's defense trait against their predator (Altwegg et al., 2006). In line, attack rates in our adaptive IGP model are modeled using sigmoidal functions (Figure 1e,f) that depend on the trait values of both species involved (Klauschies et al., 2016; van Velzen & Gaedke, 2018), which can be described as follows:

(7) $$a_{\mathrm{RP}}\left(T_{R},T_{P}\right)=a_{\text{RPmax}}∙{\left(1+e^{\left(\frac{T_{R}-T_{P}}{\sigma }\right)}\right)}^{-1}$$

(8) $$a_{\mathrm{RC}}\left(T_{R},T_{C}\right)=a_{\text{RCmax}}∙{\left(1+e^{\left(\frac{T_{C}-T_{R}}{\sigma }\right)}\right)}^{-1}$$

(9) $$a_{\mathrm{CP}}\left(T_{C},T_{P}\right)=a_{\text{CPmax}}∙{\left(1+e^{\left(\frac{T_{C}+T_{P}-1}{\sigma }\right)}\right)}^{-1}$$

where *a*_*RPmax*_, *a*_*RCmax*_, and *a*_*CPmax*_ are maximum attack rates, which can be approximately achieved when *T*_*P*_ > > *T*_*R*_, *T*_*R*_ > > *T*_*C*_, and *T*_*C*_ > > *T*_*P*_, respectively (Figure 1e,f). *σ* was assumed as 0.1 to generate moderately sharp transitions of the attack rates along the trait axes.

### 2.3 Dynamics of species traits

To describe the temporal dynamics of the species‐specific traits, we followed the approach of quantitative genetics (Abrams, 2010) in which trait values are assumed to change in the direction of the fitness gradient (Yamamichi et al., 2019). Here, fitness refers to the species *per‐capita* net growth rate, for example, the fitness of R is (1/*R*)∙(*dR*/*dt*). Trait adaptation thus enhances the fitness of the different species by allowing them to adjust their trait values in response to selection. The dynamics of *T*_*R*_, *T*_*C*_, and *T*_*P*_ are as described (cf. Klauschies et al., 2016):

(10) $$\frac{dT_{i}}{\mathrm{dt}}=v_{i}\left(\frac{\partial \frac{1}{i}\frac{\mathrm{di}}{\mathrm{dt}}}{\partial T_{i}}+B\left(T_{i},\hat{T_{i}}\right)\right)$$

where *v*_*i*_ determines the speed of trait dynamics of species *i* relative to its ecological dynamics, that is, the fitness gradient (Abrams, 2010). *v* expresses a generic ability to adaptively respond to a selective pressure, driven by adaptive phenotypic plasticity or rapid evolution (Mougi & Iwasa, 2011). Here, we refer to it as the speed of trait adaptation in line with, for example, Mougi (2012) and Klauschies et al. (2016). Finally, the boundary function *B* restricts the trait changes to their assumed biologically feasible trait ranges:

(11) $$B\left(T_{i},\hat{T_{i}}\right)=-\tan\left[\frac{\pi }{2}{\left(\frac{2}{w_{i}}\left(T_{i}-0.5\right)\right)}^{2s+1}\right]$$

where *w*_*i*_ determines the lower and upper limits of the species' biologically reasonable trait ranges (Klauschies et al., 2016). For a given *w*, the absolute range of trait changes is from 0.5‐*w*/2 to 0.5 + *w*/2. Hence, we refer to *w* as the width of trait adaptation. *s* determines the steepness of *B* close to the limits of the trait range. For the sake of brevity, we focused on scenarios where we assumed the same speed and width of trait adaptation for all three species. Hence, we simply use *v* to present *v*_*i*_ and *w* to denote *w*_*i*_ during the standard simulations. We also did a sensitivity analysis assuming that the three species have different relative speeds of trait adaptation (Figure S2), which showed that a faster adaptation of either one or two of the three species in relation to the others had little impact on species coexistence in our adaptive IGP model.

Note that all species are unable to adapt when *v* is 0. This allows us to compare species persistence and population dynamics between nonadaptive and adaptive IGP models.

### 2.4 Numerical simulation

To study the impact of trait adaptation on species coexistence, we compared the dynamics of a nonadaptive (set *v* = 0) to the dynamics of an adaptive IGP model at equilibrium conditions for a wide range of parameter values. We varied the carrying capacity *K* of R and the maximum attack rate of P on R, *a*_*RPmax*_, on a 25 × 25 grid, with 0.001 ≤ *K* ≤ 20 and 0.001 ≤ *a*_*RPmax*_ ≤ 1, as these two parameters are crucial for the long‐term behavior of the IGP system (Holt & Polis, 1997; Mylius et al., 2001). Values and meaning of the other parameters can be found in Table 1. For all simulations, initial biomass values of R, C, and P were assumed as 0.1∙*K*, 0.03∙*K*, and 0.01∙*K,* respectively, reflecting an ecologically reasonable initial pyramid biomass structure, except when we investigated the influence of initial conditions on the presence of bistability (see below). We kept all trait values at 0.5 in nonadaptive IGP models and set all initial trait values to 0.5 in the adaptive IGP models.

In order to investigate the combined impacts of *v* and *w* of trait adaptation, we varied *v* and *w* on a logarithmic grid of 25 × 25 combinations, with 0.001 ≤ *v* ≤ 0.01 and 0.01 ≤ *w* ≤ 1 (Table 1). We repeated these simulations four times using different values of *K* (2, 4, 8, and 16), allowing us to examine the effects of trait adaptation along the enrichment gradient.

Previous nonadaptive IGP models identified bistability between the exclusion of C and the exclusion of P (Holt & Polis, 1997; Mylius et al., 2001). To assess potential differences between nonadaptive and adaptive IGP modules with respect to the presence of bistability, we ran simulations of both IGP models with different initial conditions where either C dominated (*R*_*0*_ = *K*, *C*_*0*_ = 0.3∙*K*, and *P*_*0*_ = 0.01∙*K*) or both consumers had the same biomasses (*R*_*0*_ = *K*, *C*_*0*_ = 0.3∙*K*, *P*_*0*_ = 0.3∙*K*) in the parameter space defined by *K* and *a*_*RPmax*_. We extended the gradient of *K* to a maximum of 25 given that bistabilities tend to be present at high enrichment (Rogers et al., 2018). For the adaptive IGP models, we further investigated the effect of both *w* and *v* on the occurrence of bistability. Given the presence of different cases of bistability in the nonadaptive IGP model (cf. Figure 6), we conducted a bifurcation analysis by running simulations of the adaptive IGP model along the gradient of *w* under different values of *v* to gain insights into how trait adaptation affects the different attractors. We used the final biomass and trait values of the nonadaptive IGP model under the two different initial conditions to separately initialize the simulations of the adaptive IGP model with the lowest value of *w*, and then defined the new final biomass and trait values as initial values for the next simulation with higher values of *w*, iterating this process until *w* reached 1. This allowed us to stick to a certain attractor. A fairly small random perturbation (from 10^−9^ to 10^−8^) was added to the final values of the biomasses and traits before continuing the simulations with slightly higher values of *w*.

All simulations lasted for 100,000 time steps, which ensured that the biomasses and traits reached a stable or oscillating equilibrium also at high enrichment. A species is assumed to be extinct when its biomass fell below 10^−15^. Equilibrium biomasses and trait values were calculated based on the last 20,000 time steps. We considered the system to be at steady state if the coefficient of variation of biomasses was less than 0.001 or at an oscillating state if the value was greater. To better understand the biomass structure in the IGP module, we calculated the relative biomass of P with respect to the total biomass of C and P:

(12) $$\mathrm{BD}=\frac{P}{P+C}$$

For calculating this dominance index, we used the median biomasses across the last 20,000 time steps given the large oscillations at some parameter combinations. Values of *BD* of 0 and 1 indicate the exclusion of P or C from the system, respectively. C and P have the same biomasses for a value of 0.5. C dominates for *BD* < 0.5, whereas P dominates for *BD* > 0.5.

All numerical simulations were run in MATLAB, version 2018b, using solver ode23 with both absolute and relative error tolerances of 10^−10^.

## 3 RESULTS

### 3.1 Comparison of equilibrium dynamics between nonadaptive and adaptive IGP models

**FIGURE 2:**
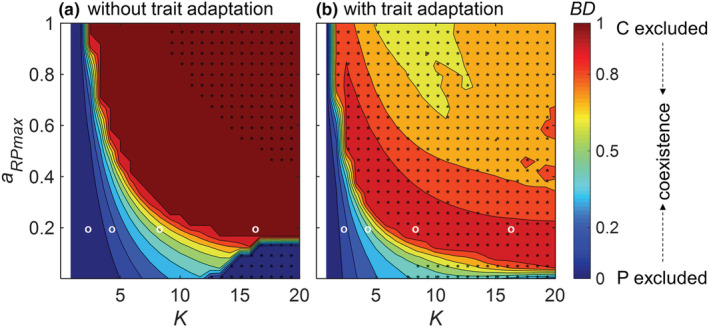
Dominance of the median biomass (*BD=P/P + C*, across the last 20,000 time steps) of the IG predator, P, over the IG prey, C, in the intraguild predation module (a) without and (b) with trait adaptation in a parameter space defined by the carrying capacity *K* and the maximum attack rate of P on the basal prey R *a*_*RPmax*_. Interpretation of the color‐coded values of *BD*: *BD* = 0, P is extinct; 0 < *BD* < 0.5, C dominates; 0.5 < *BD* < 1, P dominates; *BD* = 1, C is extinct. In the white region, C and P are both extinct at very low *K*. Regions with and without stars represent oscillatory or steady states, respectively. White circles mark the parameter combinations with *K* equal to 2, 4, 8, and 16 and *a*_*RPmax*_ of 0.2 used in Figures 3, 4, 5 and S6–S7. The width and the speed of trait adaptation were chosen as *w* = 0.3 and *v* = 0.01, respectively. Other parameter values are given in Table 1

In our nonadaptive IGP model, coexistence of all three species was limited to a narrow parameter space (Figure 2a, S3). At very low carrying capacity *K*, neither the IG prey C nor the IG predator P could persist with the basal prey R due to the low biomass of R (white region in Figure 2a). C excluded P at somewhat higher *K* (dark blue in Figure 2a), whereas P excluded C in reverse at high *K* unless the maximum attack rate of P on R, *a*_*RPmax*_ was very small (dark red in Figure 2a). Only at intermediate *K*, all three species coexisted, in particular at lower values of *a*_*RPmax*_. The biomasses of R and P generally increased with increasing *K*, whereas C tended to increase first and then declined (Figure S3). Within the region of stable coexistence of all three species, C was the superior competitor in exploiting R, that is, the flux from R to C was substantially higher than the flux from R to P (Figure S4a).

Trait adaptation greatly enhanced species persistence in our IGP model (Figure 2b, S5). The region where only C persisted with R, whereas P was excluded was strongly compressed in the adaptive IGP model (Figure 2b). Except for a very small region at very low *K* where both C and P were excluded, all three species coexisted either at a steady or at a oscillatory state. At lower values of *K*, the biomass dominance of P gradually increased with *a*_*RPmax*_, whereas at higher values of *K*, it first increased and after passing a particular threshold value decreased with a further increase of *a*_*RPmax*_ (Figure 2b, S5a). The opposite pattern holds true for C. Consistent with the nonadaptive model, the flux from R to C was mostly higher than the flux from R to P (*F*_*RP*_) in the adaptive model, whereas the flux from C to P was mostly comparable with *F*_*RP*_ in a large parameter space (Figure S4b). Species showed different trait expressions in the parameter space (Figure S5c). Specifically, at lower values of both *K* and *a*_*RPmax*_, R defended against C, in turn, C offended against R, and P offended against C. By contrast, at higher values of *K* and *a*_*RPmax*_, R slightly defended against P, whereas C defended against P, and P increased its offense against the defense of R.

### 3.2 Effects of the width and speed of trait adaptation on IGP dynamics along the enrichment gradient

**FIGURE 3:**
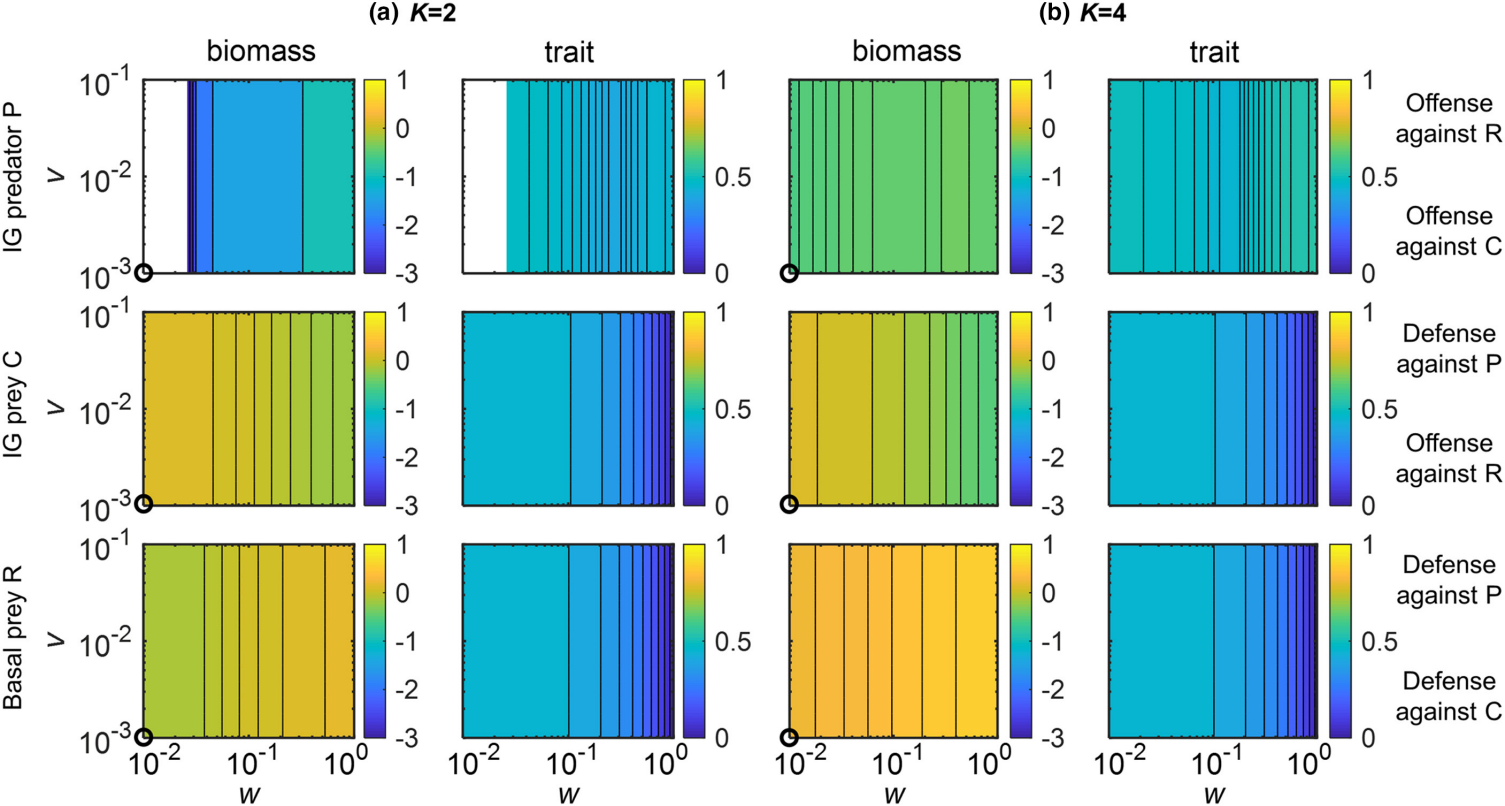
Effects of the width *w* and the speed *v* of trait adaptation on log_10_(biomasses), and trait values of the basal prey R (third row), the IG prey C (second row) and the IG predator P (first row) in the adaptive intraguild predation model with a carrying capacity of (a) *K =* 2 (the first two left columns) and (b) *K* = 4 (the last two right columns). Biomasses and trait values are average values across the last 20,000 time steps. White regions indicate that the species of the respective panel is excluded from the system. Black circles in biomasses approximately indicate the species log_10_(biomasses) in the nonadaptive IGP model shown in Figure 2a. The maximum attack rate of P on the basal prey R *a*_*RPmax*_ was chosen as 0.2. Other parameter values are given in Table 1

**FIGURE 4:**
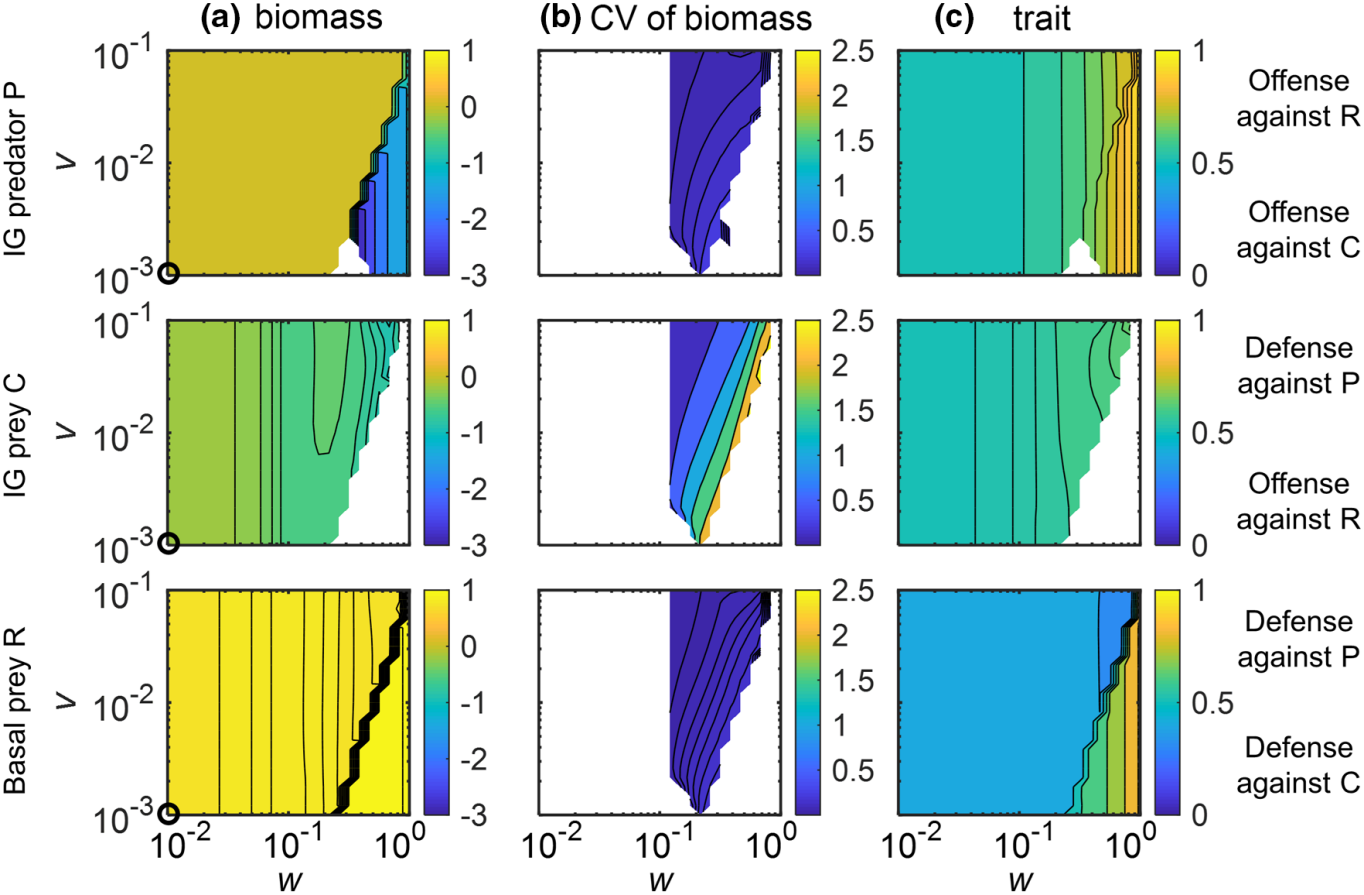
Effects of the width *w* and the speed *v* of trait adaptation on the log_10_(biomasses), coefficient of variation (CV) of biomasses, and trait values of the basal prey R, the IG prey C, and the IG predator P in the adaptive intraguild predation model with a carrying capacity *K* of 8. Biomasses and trait values are average values across the last 20,000 time steps. White regions in (a,c) indicate that the species of the respective panel is excluded from the system and (b) additionally indicate stable equilibrium (CV < 0.001). Black circles approximately indicate the species biomasses in the nonadaptive intraguild predation model shown in Figure 2a. The maximum attack rate of P on the basal prey R *a*_*RPmax*_ was chosen as 0.2. Other parameter values are given in Table 1

**FIGURE 5:**
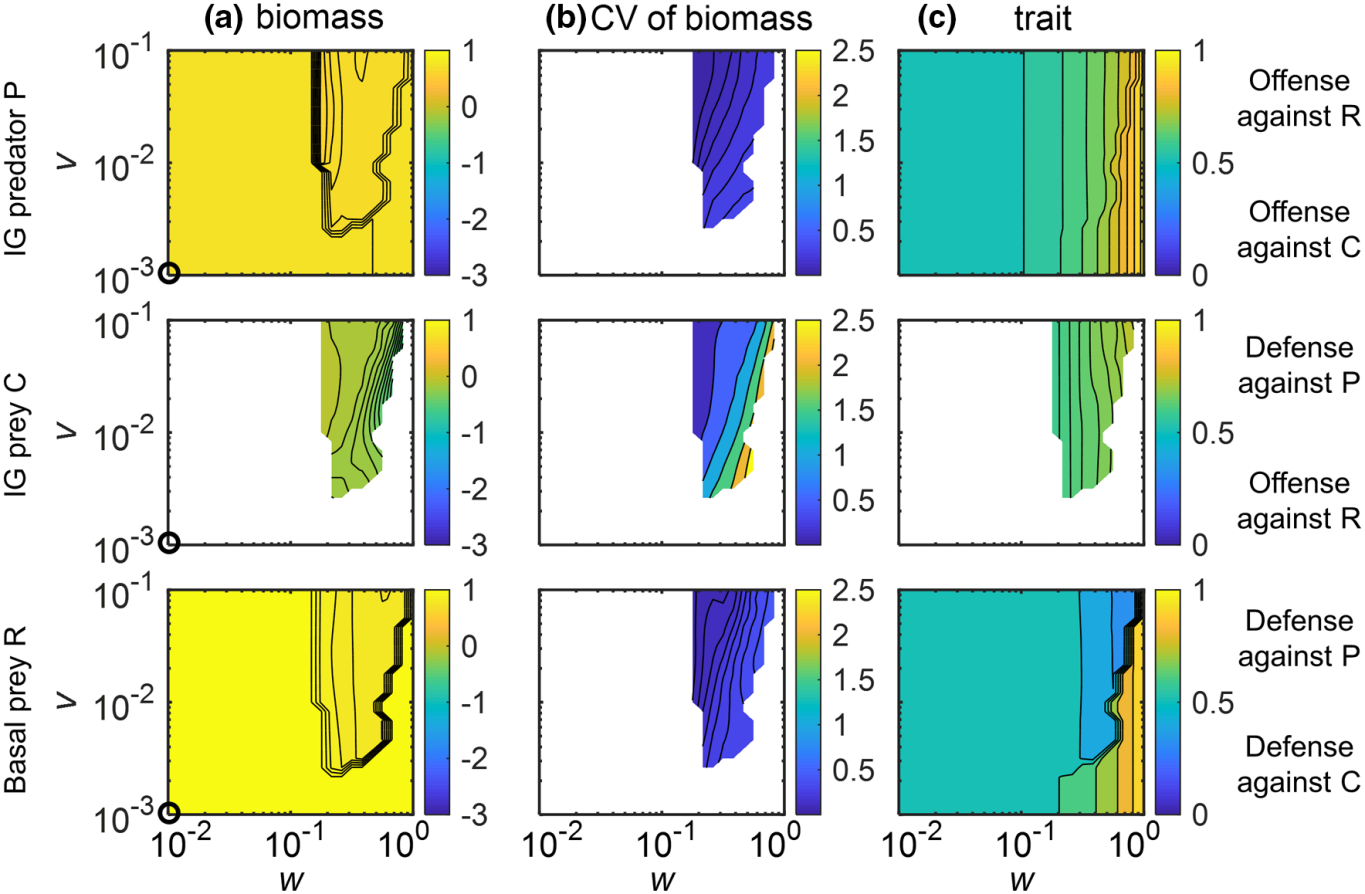
Effects of the width *w* and the speed *v* of trait adaptation on the dynamics of the adaptive intraguild predation module with a carrying capacity *K* of 16. Interpretations are the same as in Figure 4

As the nonadaptive IGP model predicted gradual changes in the equilibrium states along *K* (Figure 2a), we selected four parameter combinations in which this model showed qualitative differences with respect to species persistence and biomass structure (white circles in Figure 2; for dynamics in time series see Figures S6 and S7: (1) *K* = 2, R persisted with C, whereas P was extinct; (2) *K* = 4, R, C, and P coexisted, and C had a higher biomass than P; (3) *K* = 8, again all species coexisted, but C and P had similar biomasses; (4) *K* = 16, R persisted with P, whereas C was extinct). For each case, we simulated the dynamics of the adaptive IGP model for different values of the speed *v* and the width *w* of trait adaptation (Figures 3, 4, 5).

In contrast to the nonadaptive model version, all three species coexisted at *K* = 2 as soon as a little trait adaptation was allowed for (*w* > 0.026 in Figure 3a). This holds even more for *K* = 4 where an increase in *w* further stabilized the coexistence of all three species as the biomasses of P and C became more similar (Figure 3b, S8). For both *K* = 2 and 4, the coexistence of all three species was always associated with a stable equilibrium of the trait and biomass dynamics. Additionally, as a consequence of trophic cascading, the higher top‐down control of C by P associated with higher values of *w* partly released R from top‐down control by C, resulting in a higher biomass of R (Figure 3, S9). The equilibrium trait values indicate that C adapted to offend against R and in turn, R adapted to defend against C, whereas P did not strongly offend against any specific prey due to the mostly small biomass difference between R and C (Figure 3).

At higher enrichment (*K* = 8 and 16), the effects of trait adaptation on the population dynamics of the IGP system were more complex compared with lower enrichment (Figures 4 and 5). At *K* = 8, trait adaptation destabilized species coexistence at high *w* and low *v* as C did not persist (Figure 4). Additionally, the extinction of C strongly reduced the biomass of P and slightly increased the biomass of R. For *K* = 16, trait adaptation promoted coexistence of all three species for constrained widths (ca. 0.2 < *w* < 0.7) and sufficiently high speeds (*v* > 0.03) of trait adaptation (Figure 5). Taken all results together, increasing *w* always promoted the coexistence of the three species at lower *K*, whereas this only holds for a constrained *w* that was neither too small nor too large at higher levels of *K*.

In addition, for *K* = 8, when all three species coexisted, the corresponding biomass and trait values settled down at a steady state when *w* was rather low (*w* < 0.12) or approached an oscillating state when *w* was higher (ca. 0.12 < *w* < 0.8) (Figure 4). By contrast, for *K* = 16, the three species always coexisted at oscillatory states (Figure 5). Independent of the actual value of *K*, the temporal variability of biomasses indicated by the coefficient of variation (CV) of all species gradually decreased with increasing *v*, and the CV of the biomass of C was typically much higher than that of the other two species (Figures 4 and 5).

The patterns in trait values of the three species were similar at *K* = 8 and 16 (Figures 4 and 5). Trait changes of R clearly followed the trade‐off between defense against C or P, that is, R greatly increased its defense against P after the exclusion of C. C showed a defense against the predation pressure of P, which was more obvious at *K* = 16. P adapted by increasing its trait value to offend against the profitable R given the high biomass of R compared with C, and the strength of offense increased after the exclusion of C. Within the region of coexistence of all three species at oscillatory states, the trait values of all species changed more pronouncedly at higher values of *w* independent of *v* (Figure S10).

### 3.3 Effects of trait adaptation on the presence of bistability in the IGP module

**FIGURE 6:**
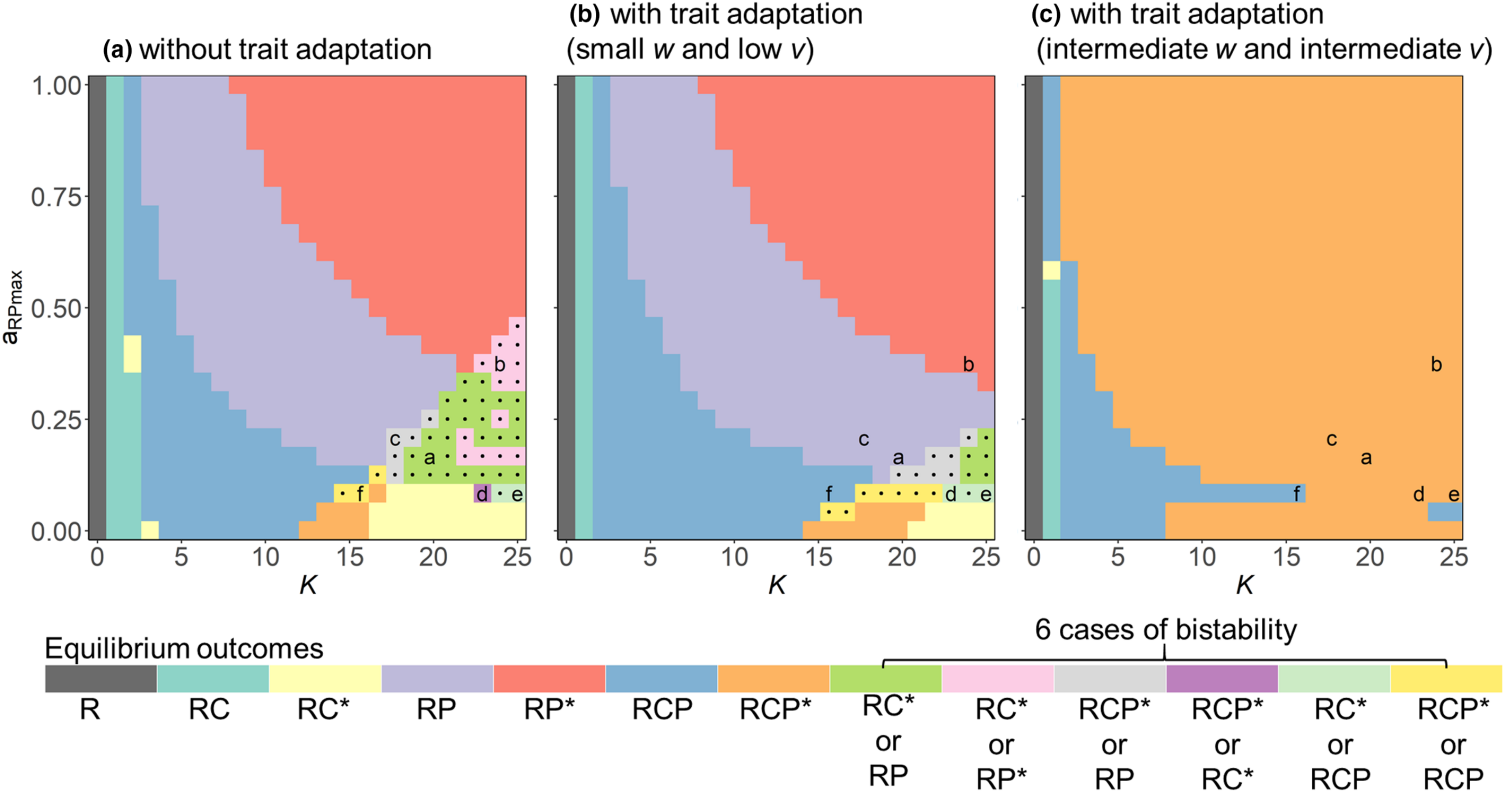
Equilibrium outcomes of the (a) nonadaptive and (b,c) adaptive intraguild predation models in a parameter space defined by the carrying capacity (*K*) and the maximum attack rate of IG predator, P, on the basal prey, R (*a*_*RPmax*_). *w* and *v* denote the width and the speed of trait adaptation, respectively, which were assumed as *w* = 0.04 and *v* = 0.01 in (b), and *w* = 0.3 and *v* = 0.01 in (c). Other parameter values are given in Table 1. Dots mark regions of bistability. Labels of a–f represent the six parameter combinations between *K* and *a*_*RPmax*_ resulting in the six cases of bistability used in Figure S13–S15. Abbreviations of equilibrium outcomes: R, only R persists; RC, R and the IG prey C coexist; RP, R and P coexist; RCP, coexistence of all three species. Equilibria marked with * denote oscillating

Using different initial conditions, the nonadaptive IGP model displayed six cases of bistability, in particular at higher *K* and lower *a*_*RP*max_ (Figure 6a), for example, between the exclusion of P and the exclusion of C, and between coexistence of C and P and P excluding C, or C excluding P. We also found bistability between final oscillatory and steady states with the same species persisting, which was insensitive to the threshold we used to distinguish between static or oscillatory states (Figure S11).

The region of bistability was already strongly reduced when assuming very small values of *w* and *v* (Figure 6b, S12a), and there was no case of bistability at intermediate values of *w* independent of *v* (Figure 6c, S12b,e,h). Rather, the three species coexisted either at an oscillatory or at a steady state. Although there was also no sign of bistability at higher values of *w* (Figure S12c,f,i), the coexistence of all three species was only possible when the value of *v* was sufficiently high.

Regarding the six cases of bistability present in the nonadaptive IGP model, we further conducted a bifurcation analysis by running simulations of the adaptive IGP model along the gradient of *w* under different levels of *v* (Figure S13–S15). We found that trait adaptation strongly reduced the occurrence of bistability and a higher value of *v* generally reduced the value of *w* required to suppress bistability, thereby often promoting an equilibrium state with all three species coexisting. The influence of *w* and *v* on the occurrence of bistability further depended critically on *K* and *a*_*RPmax*_. Illustrating this for a combination of *K* and *a*_*RPmax*_, the nonadaptive IGP model either showed coexistence of R and C at an oscillatory state or coexistence of R and P at a steady state (Figure S14a). In the adaptive IGP model, the oscillatory R‐C attractor turned into an oscillatory R‐C‐P attractor, whereas the steady state R‐P attractor still existed at very low *w*. For somewhat higher values of *w* (*w* > 0.03), the observed bistability vanished and only the steady state R‐P attractor remained. While this attractor only remained for lower and very high values of *w*, it enabled coexistence of all three species for at intermediate *w* values (0.2 < *w* < 0.7). Similarly complex patterns arose for the bistability cases described in Figure S14b,d,e, except that the two different attractors established in the nonadaptive IGP model still existed at very low *w*. Other cases of bistability disappeared once the IGP module became adaptive (Figure S14c,f).

## 4 DISCUSSION

We developed an adaptive IGP model that allowed all species to coadjust their defense or offense strategies in response to the prevailing selection regime arising from the complex trophic interactions. This goes beyond previous IGP models considering constant trait values or trait adaptation only within individual species. We showed that a larger width of trait adaptation within all species facilitated species coexistence independent of the speed of trait adaptation at lower enrichment, while this effect occurred only for a constrained width and fast trait adaptation at high enrichment. Generally, trait adaptation strongly promoted the occurrence of oscillatory dynamics, providing a mechanism of species coexistence arising from the fluctuations in biomasses and traits. Under equilibrium conditions, trait adaptation facilitated coexistence of all three species by enabling adjustment to suitable trait values. Remarkably, trait adaptation strongly reduced the occurrence of bistability encountered in the nonadaptive IGP model and greatly promoted the attractor with all three species coexisting. These findings demonstrate that coadaptation driven by both competitive and predator–prey interactions may substantially contribute to the maintenance of IGP modules in natural food webs.

### 4.1 Trait adaptation promotes coexistence of all three species

Rather than general defenses and offenses, we assumed bidirectional trait axes for the basal prey R and IG predator P, that is, predator‐specific defense traits or prey‐specific offense traits, which are known to play a major role in facilitating species coexistence (Ikegawa et al., 2015; Nakazawa et al., 2010; van Velzen, 2020). For instance, R reallocated its species‐specific defense after the exclusion of the IG prey C by shifting from a slight defense against predation by C to a strong defense against P (Figures 4 and 5). The ability of P to adjust its offense according to the altered availability of R and C also broadened the parameter range of species coexistence in line with previous studies (Chattopadhyay et al., 2015; Křivan & Diehl, 2005). At lower enrichment, C was highly competitive but a large width of trait adaptation enabled P to strongly increase its offense for R promoting its persistence (Figure 3). This process was independent of the speed of trait adaptation in line with Nakazawa et al. (2010).

Previous studies of nonadaptive IGP models have shown that C tended to be very fragile once P persisted at higher enrichment (Holt & Polis, 1997; Mylius et al., 2001). We found that processes mechanistically resembling indirect evolutionary rescue (Yamamichi & Miner, 2015) profoundly reduced the extinction of C in our adaptive IGP model. When C fell to a very low abundance at higher enrichment, P increased its offense against R to better exploit the most abundant prey R. Thus, R reduced its defense against C in favor of defending against P. This allowed C to decrease its defense against P and to offend more against R which was also less protected against consumption by C. These processes reduced the losses and increased food availability of C, enabling its recovery from low densities. Overall, C mostly offended against R at lower enrichment and defended against P at higher enrichment (Figure S5) which is consistent with findings from a nonadaptive model where C exerted a high competition pressure on P at low enrichment but faced a high predation pressure at high enrichment (Novak, 2013). A sufficiently large and fast trait adaptation was needed for C to persist at higher enrichment (Figures 4 and 5). However, trait adaptation over a very large width resulted in high‐amplitude biomass oscillations that may lead to biomass values of C below the extinction threshold. This extinction risk was strongly reduced with increasing speed of trait adaptation. Additionally, in line with previous studies (Vasseur et al., 2011; Yamamichi et al., 2022), biomass‐trait feedbacks drive species trait values to vary over time at higher enrichment, which creates new opportunities for coexistence that do not exist for any fixed trait values (Figure S16). Thus, in the adaptive IGP model, coexistence was strongly promoted as species were able to adapt to suitable trait values at a steady state enabling, for example, indirect evolutionary rescue. Furthermore, time‐varying trait values continuously alter the selection regime at oscillatory states, giving rise to fluctuation‐dependent mechanisms of coexistence (Abrams, 2006; Chesson, 2000). In both cases, trait adaptation releases rare species from predation and competition pressure at low densities and thereby induces negative frequency dependent selection that stabilizes species coexistence (Klauschies et al., 2016; van Velzen, 2020).

A striking number of examples from field studies and experiments reveal that trait adaptation occurring on timescales similar to those of ecological dynamics are common, with potentially dramatic impacts on population dynamics and community structure (Becks et al., 2012; Hiltunen et al., 2014; Irwin et al., 2015; Rogers et al., 2018; Yoshida et al., 2003). Accounting for the ubiquitous potential of coadaptation among all species provides a mechanism of how IGP can be common in nature and thus resolves the contradiction between the empirical evidence of the widespread occurrence of IGP (e.g., Arim & Marquet, 2004) and the theoretical predictions that IGP should only occur under restricted conditions (e.g., Holt & Polis, 1997). Given that IGP modules are normally embedded within more complex food webs (Stouffer & Bascompte, 2010; Wang et al., 2019), it would be valuable to extend our adaptive model to food webs including higher trophic levels to deepen our understanding of more complex natural food webs.

In line with classical predator–prey theory (Rosenzweig, 1971), enrichment tended to destabilize the population dynamics in our nonadaptive IGP model with large amplitude oscillations arising at higher levels of enrichment (Figure 2a). For most parameter combinations, the biomass of C had larger oscillations than that of R and P.

Although Mougi and Nishimura (2008) suggested that trait adaptation may reduce the temporal variability of population dynamics and thereby result in a possible resolution of the paradox of enrichment, we found that population dynamics in the adaptive IGP model were already destabilized at lower enrichment levels than in the nonadaptive model (Figure 2). Hence, while trait adaptation may generally destabilize the population dynamics in an IGP model, it may at the same time buffer the amplitudes that are generated solely by the ecological interactions. In this way, our results add to a growing body of research (de Andreazzi et al., 2018; Kondoh, 2003; Nakazawa et al., 2010; Vasseur et al., 2011; Yamauchi & Yamamura, 2005), showing that rapid coadaptation among prey's defense and predator's offense traits may buffer already existing oscillations in the species biomasses and thereby help to resolve the paradox of enrichment in IGP systems. The dampening effect of trait adaptation at higher enrichment levels relied on sufficiently fast trait adaptation (Figures 4 and 5) and likely resulted from reduced time lags in the adjustments toward currently optimal trait values (Klauschies et al., 2016). By contrast, the destabilization effect of trait adaptation at lower enrichment levels may partly arise from a different food web structure as coexistence of all three species was often only possible with ongoing trait adaptation (Figure S16).

### 4.2 Trait adaptation reduces the presence of bistability of the IGP module

Previous studies showed that nonadaptive IGP models often exhibit bistability (Holt & Polis, 1997; Mylius et al., 2001). When R exhibits logistic growth and the two consumers have linear functional responses, the corresponding IGP model has only one case of bistability, that is, either C or P persists with R (Holt & Polis, 1997). When assuming a flow through system and linear or saturating functional responses for the consumers, an additional combination of bistability, either the coexistence of the three species or the exclusion of C, occurred (Verdy & Amarasekare, 2010). Our nonadaptive IGP model, with logistically growing R and saturating functional responses of the consumers, exhibited more cases of bistability in a large parameter space, for example, either the coexistence or the exclusion of P, and either coexistence at a steady or oscillatory state. Such bistabilities imply that the nonadaptive IGP module is highly unpredictable and prone to regime shifts, for example, by seemingly minor pulse perturbations as they occur frequently in nature (Beauchesne et al., 2021; Drury & Lodge, 2009; McCormick et al., 2019).

Adaptive systems could buffer such perturbations better than nonadaptive ones and rapid trait adaptation may increase the resilience and the elasticity of predator–prey systems (Raatz et al., 2019; Wojcik et al., 2021). So far, the effect of trait adaptation on alternative states of an IGP module has been less explored, but see Patel and Schreiber (2015), who found that changes in phenotypes of consumers in the IGP module can alter population biomasses and thus may drive the system from one alternative state to another. We show that the likelihood of bistability was strongly reduced once trait adaptation was introduced into the IGP module and even vanished when the width or the speed of trait adaptation was sufficiently large and rapid (Figure 6 and S12).

Overall, using general IGP models without and with trait adaptation only based on a few widely accepted assumptions, we found that trait adaptation generally facilitated species coexistence when all species were able to adapt their trait values in response to selection, though different widths of trait adaptation were needed along the gradient of enrichment. Furthermore, trait adaptation strongly reduced the presence of bistability making the system more robust against perturbations. Thus, we demonstrate that intraspecific trait adaptation through adaptive phenotypic plasticity or rapid evolution can promote species persistence and stabilize food web dynamics. This leads us to pay more attention to the conservation of the species' potential to adapt their traits in response to an altered environment and thus, more generally, to maintain intraspecific functional diversity in natural ecosystems, especially under the increasing threat of homogenization due to environmental changes and intense human activities (Park & Razafindratsima, 2019; Zwiener et al., 2018).

## AUTHOR CONTRIBUTIONS

**Xiaoxiao Li:** Conceptualization (supporting); data curation (lead); formal analysis (lead); investigation (lead); methodology (supporting); software (lead); visualization (lead); writing – original draft (lead). **Toni Klauschies:** Conceptualization (equal); data curation (equal); formal analysis (equal); investigation (equal); methodology (lead); software (equal); writing – review and editing (equal). **Wei Yang:** Formal analysis (equal); funding acquisition (equal); investigation (equal); project administration (equal); supervision (equal); visualization (equal); writing – review and editing (equal). **Zhifeng Yang:** Project administration (equal); supervision (equal); visualization (equal); writing – review and editing (equal). **Ursula Gaedke:** Conceptualization (equal); formal analysis (equal); funding acquisition (equal); investigation (equal); methodology (equal); project administration (equal); supervision (equal); validation (equal); writing – review and editing (equal).

## CONFLICT OF INTEREST

All authors declare no conflict of interest.

## Supporting information


Figure S1‐S16

## Data Availability

Data available from the Dryad Digital Repository: https://doi.org/10.5061/dryad.mw6m9060b (Li et al., 2023).
