# Supplementary material for: Trait adaptation enhances species coexistence and reduces bistability in an intraguild predation module
Source: Ecol Evol. 2023 Jan 23;13(1):e9749. doi: 10.1002/ece3.9749 (PMC9871339; doi:10.1002/ece3.9749)
Supplement: Supplementary file 1 — Figure S1‐S16 [file ECE3-13-e9749-s001.docx]

**Supporting information**

**Trait adaptation enhances species coexistence and reduces bistability in an intraguild predation module**

Xiaoxiao Li^1,2,3^, Toni Klauschies^4^, Wei Yang^2,5*^, Zhifeng Yang^1,2,3^, Ursula Gaedke^4^

^1^Guangdong Provincial Key Laboratory of Water Quality Improvement and Ecological Restoration for Watersheds, School of Ecology, Environment and Resources, Guangdong University of Technology, Guangzhou 510006, China

^2^State Key Laboratory of Water Environment Simulation, School of Environment, Beijing Normal University, Beijing 100875, China

^3^Southern Marine Science and Engineering Guangdong Laboratory (Guangzhou), Guangzhou 511458, China

^4^Department of Ecology and Ecosystem Modelling, Institute of Biochemistry and Biology, University of Potsdam, Potsdam 14469, Germany

^5^Yellow River Estuary Wetland Ecosystem Observation and Research Station, Ministry of Education, Shandong 257500, China

***Corresponding author:** Prof. Wei Yang, E-mail address: yangwei@bnu.edu.cn, Postal address: 19 Xinjiekouwai St., Haidian District, 100875, Beijing, China

**Supplementary figures**

**
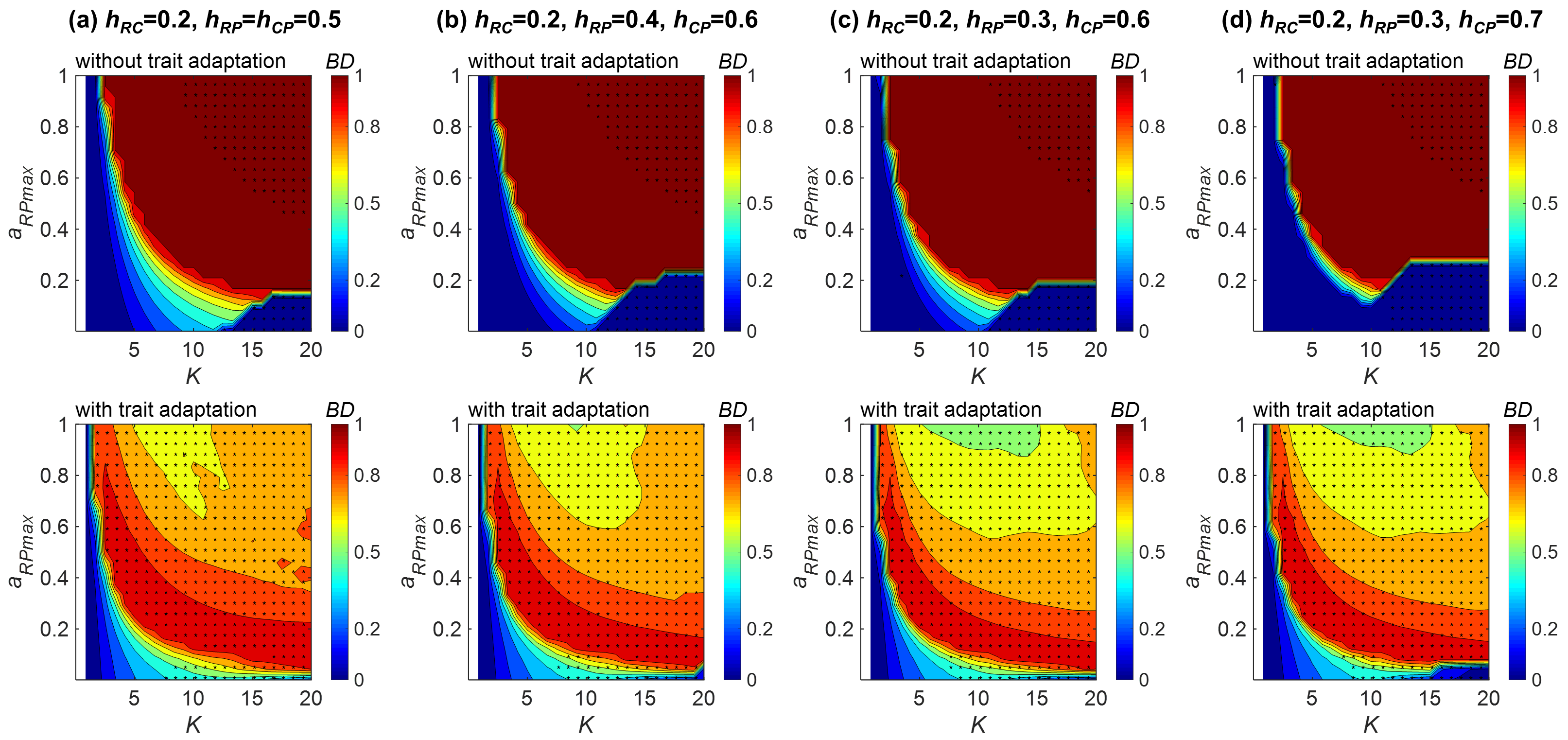
**

**Figure S1** Dominance of the median biomass (*BD=P/P+C*, across the last 20,000 time steps) of the IG predator, P, over the IG prey, C, in the non-adaptive (upper row) and adaptive (lower row) intraguild predation models with different handling times of P feeding on the basal prey R and C in the parameter space defined by the carrying capacity *K* and the maximum attack rate of P on the basal prey R *a_RPmax_*. *h_ij_* is the handling time of species *j* feeding on species *i*. Interpretation of the color-coded values of *BD*: *BD* = 0, P is extinct; 0 < *BD* < 0.5, C dominates; 0.5 < *BD* < 1, P dominates; *BD* = 1, C is extinct. In the white region, C and P are both extinct at very low *K*. Regions with and without stars represent oscillatory or steady states, respectively.

**
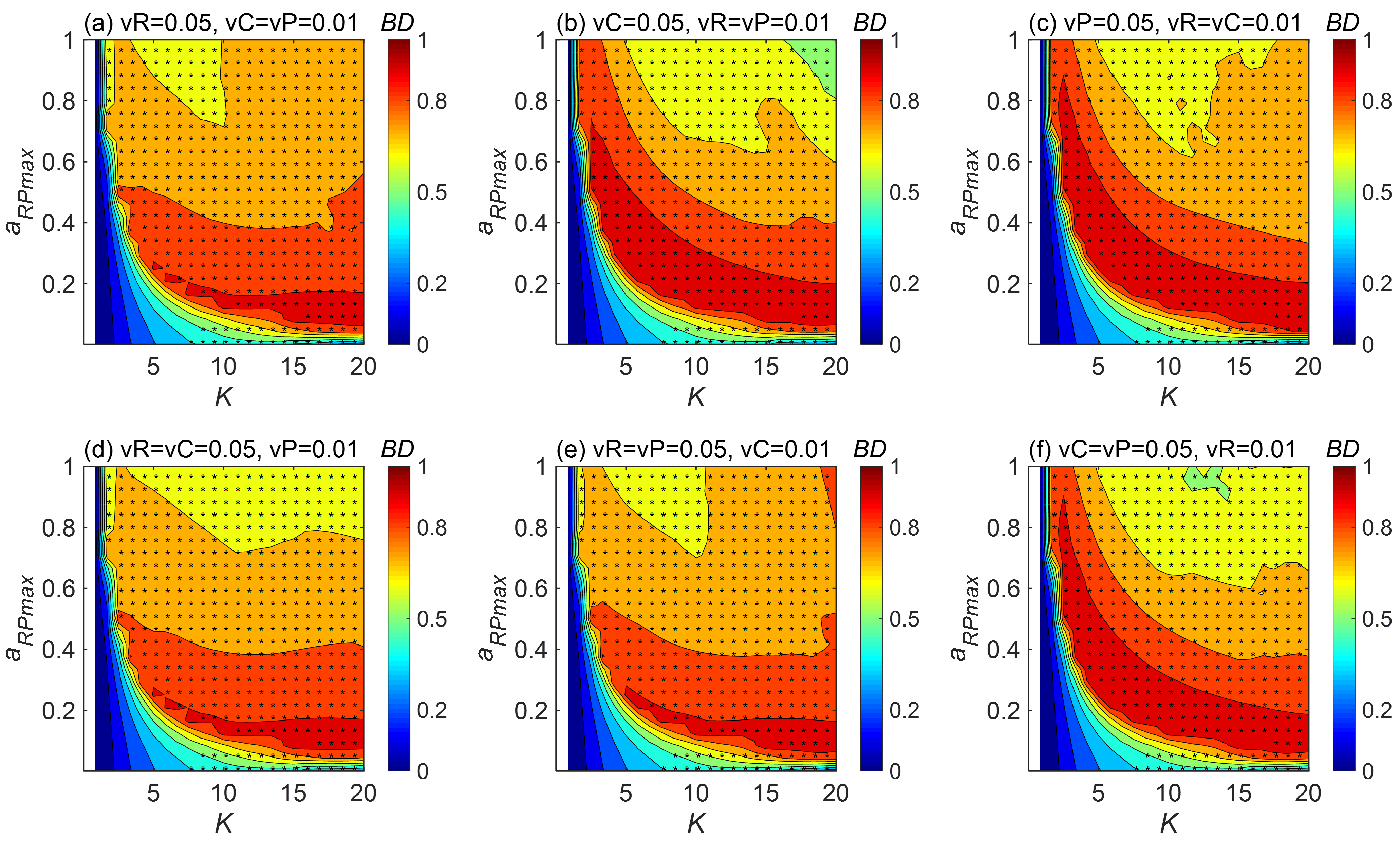
**

**Figure S2** Dominance of the median biomass (*BD=P/P+C*, across the last 20,000 time steps) of the IG predator (P) over the IG prey (C) in the adaptive intraguild predation models with different relative speeds of trait adaptation in the parameter space defined by the carrying capacity *K* and the maximum attack rate of P on the basal prey R *a_RPmax_*. vR, vC, and vP denote the speed of trait adaptation of the basal prey, the IG prey, and the IG predator, respectively. Interpretation of the color-coded values of *BD*: *BD* = 0, P is extinct; 0 < *BD* < 0.5, C dominates; 0.5 < *BD* < 1, P dominates; *BD* = 1, C is extinct. In the white region, C and P are both extinct at very low *K*. Regions with and without stars represent oscillatory or steady states, respectively.


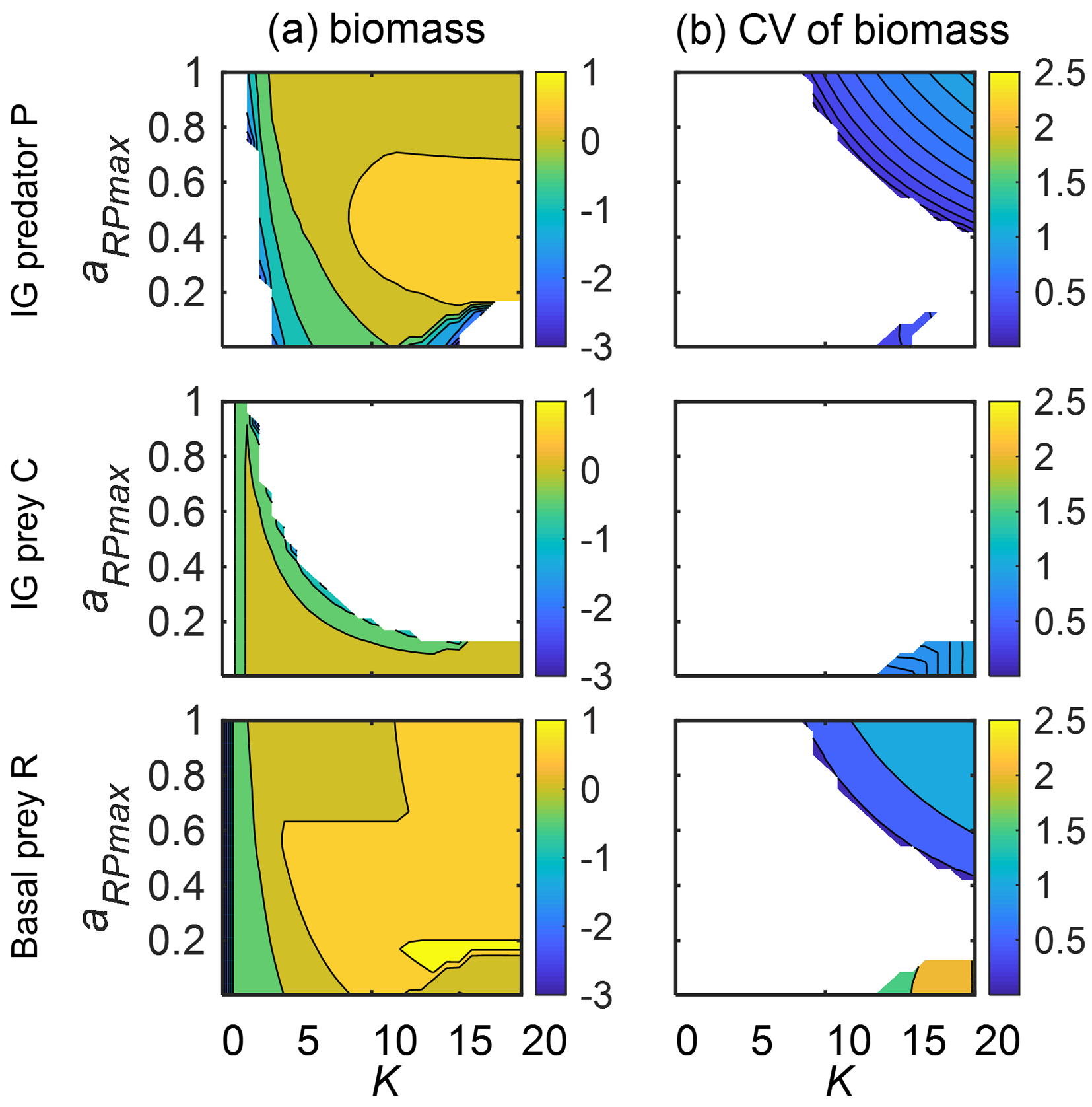


**Figure S3** (a) log_10_(biomasses) and (b) coefficient of variation (CV) of biomasses of the basal prey R, the IG prey C, and the IG predator P in the non-adaptive intraguild predation module in a parameter space defined by the carrying capacity *K* and the maximum attack rate of P on the basal prey R *a_RPmax_*. White regions indicate in (a) the exclusion of the species of the respective panel and in (b) stable equilibrium (CV < 0.001).


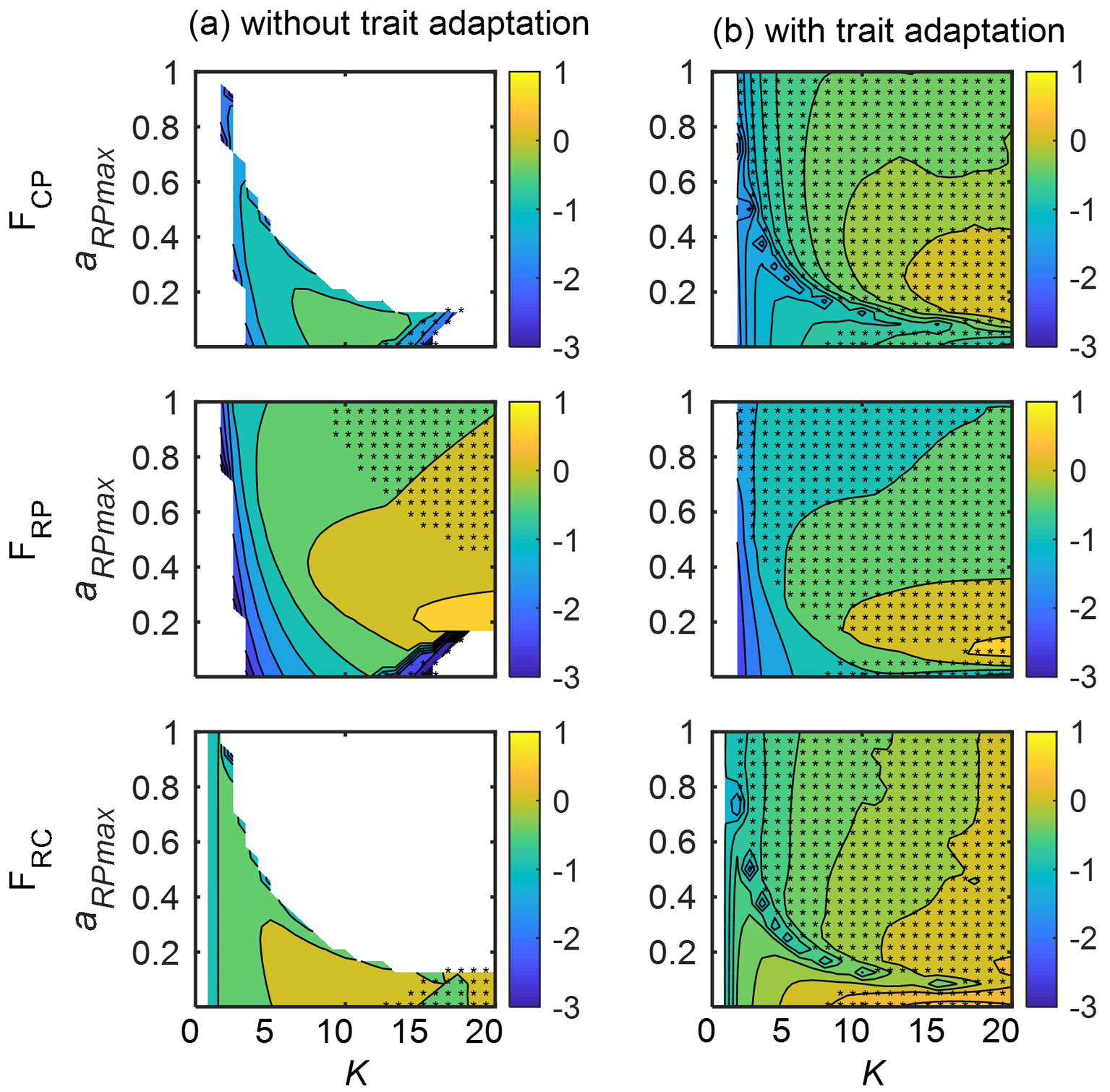


**Figure S4** log_10_(Fluxes) from the basal prey to the IG prey F_RC_, from the basal prey to the IG predator F_RP_, and from the IG prey to the IG predator F_CP_ in the (a) non-adaptive and (b) adaptive intraguild predation module in the parameter space defined by the carrying capacity *K* and the maximum attack rate of the IG predator on the basal prey *a_RPmax_*. White regions indicate the exclusion of either the prey or the predator related to the flux. Regions with and without stars represent oscillatory or steady states, respectively. The width *w* and the speed *v* of trait adaptation were chosen as *w* = 0.3 and *v* = 0.01, respectively.


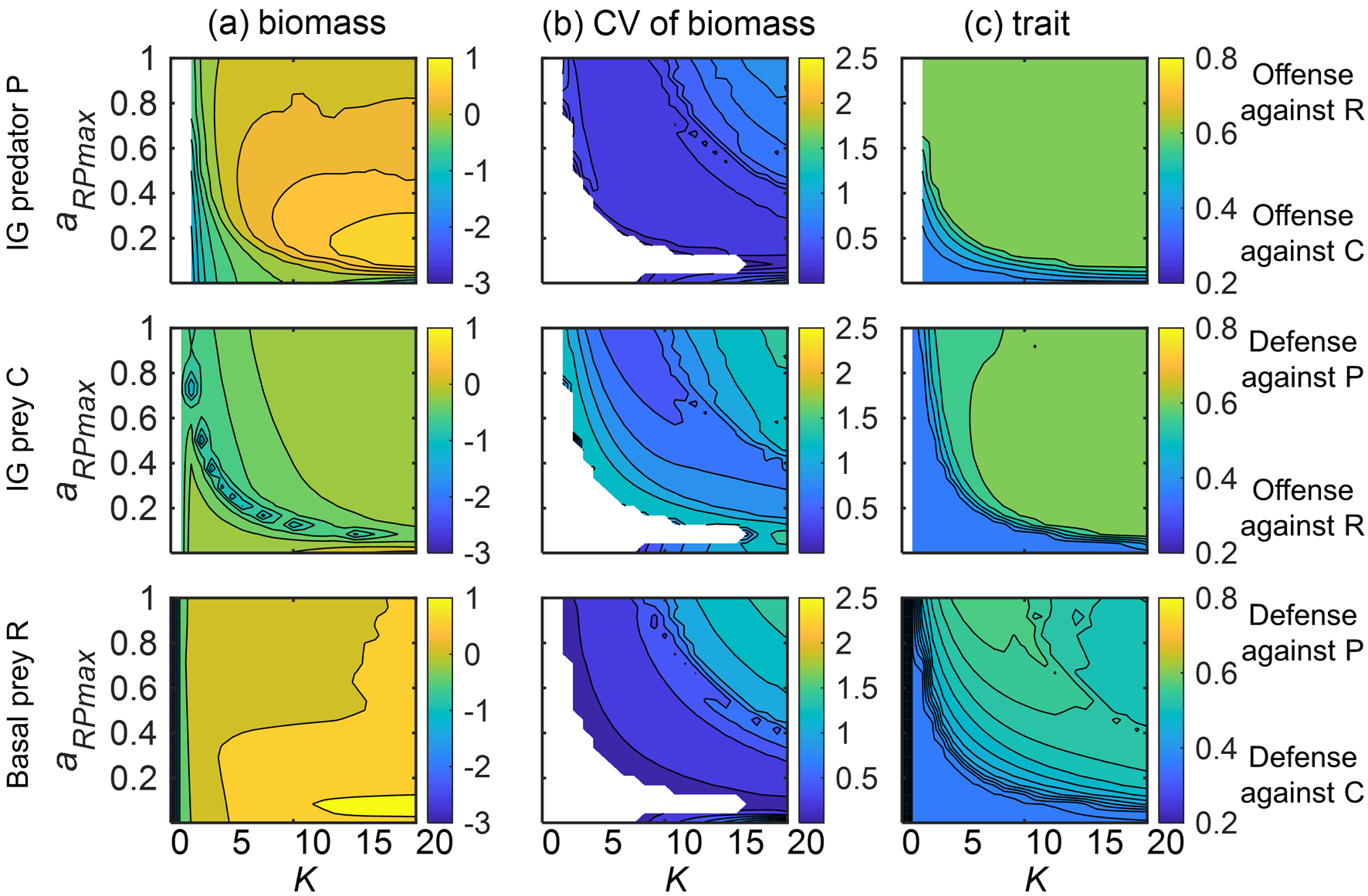


**Figure S5** (a) log_10_(Biomasses), (b) coefficient of variation (CV) of biomasses and (c) trait values of the basal prey R, the IG prey C, and the IG predator P in the adaptive intraguild predation module in the parameter space defined by carrying capacity *K* and the maximum attack rate of P on the basal prey R *a_RPmax_*. White regions indicate in (a, c) the exclusion of the species of the respective panel and in (b) stable equilibrium (CV < 0.001). The width *w* and the speed *v* of trait adaptation in the adaptive intraguild predation model were chosen as *w* = 0.3 and *v* = 0.01, respectively.


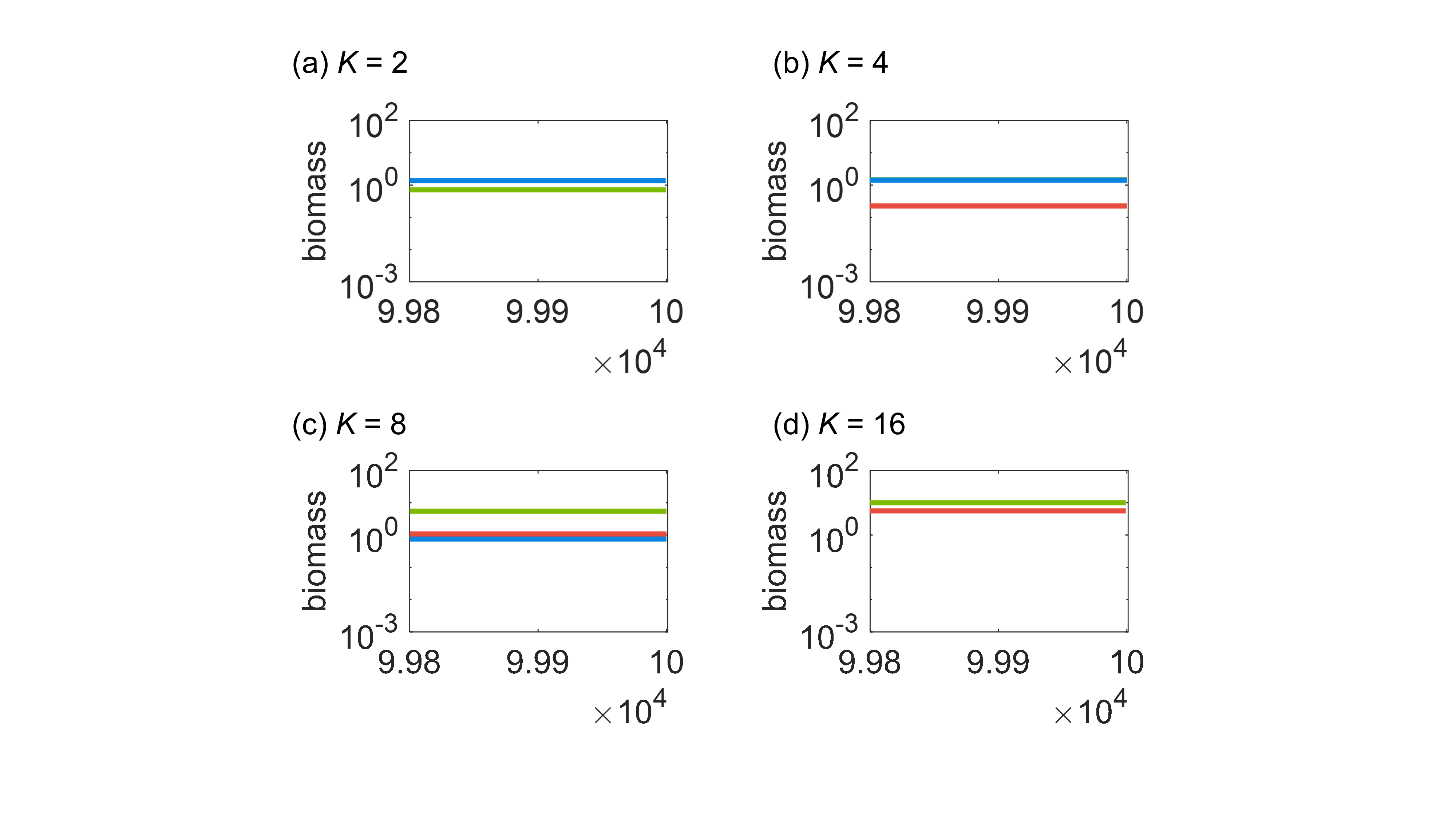


**Figure S6** Biomass dynamics of the basal prey (green line), the IG prey (blue line), and the IG predator (red line) in the non-adaptive intraguild predation model at different levels of enrichment represented by the carrying capacity (*K*): (a) *K* = 2, (b) *K* = 4, (c) *K* = 8, and (d) *K* = 16.


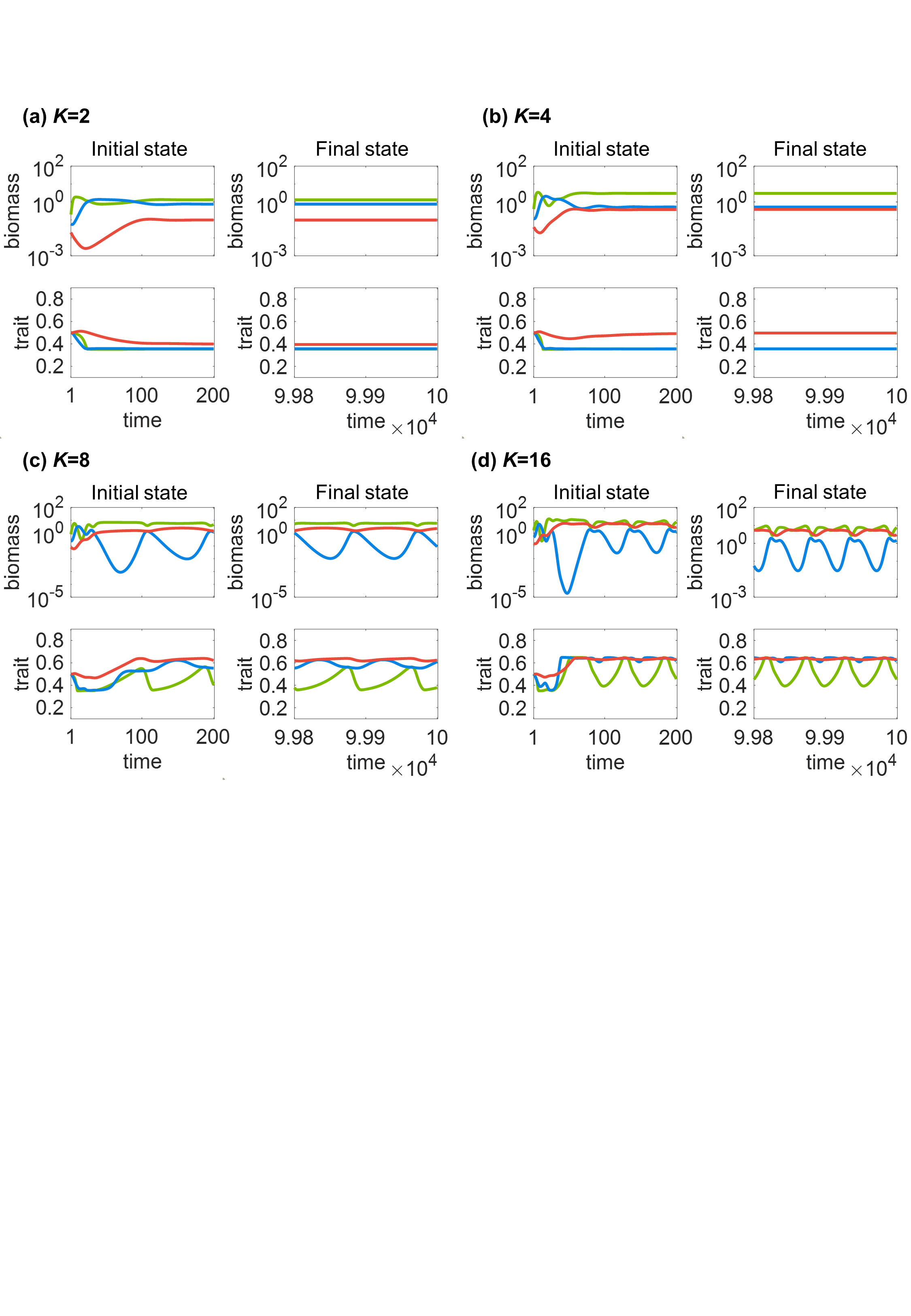


**Figure S7** Biomass and trait dynamics for adaptive intraguild predation models at different levels of enrichment represented by the carrying capacity (*K*): (a) *K* =2, (b) *K* = 4, (c) *K* = 8, and (d) *K* = 16, showing the initial (left panels, first 200 time steps) and final (right panels, last 200 time steps) states. Green, blue, and red colors indicate the basal prey, the IG prey, and the IG predator in the IGP module, respectively. The width (*w*) of trait adaptation was assumed as 0.3, indicating that species can change their trait values from 0.35 (0.5-*w*/2) to 0.65 (0.5+*w*/2). The speed of trait adaptation for each species was assumed as 0.01.


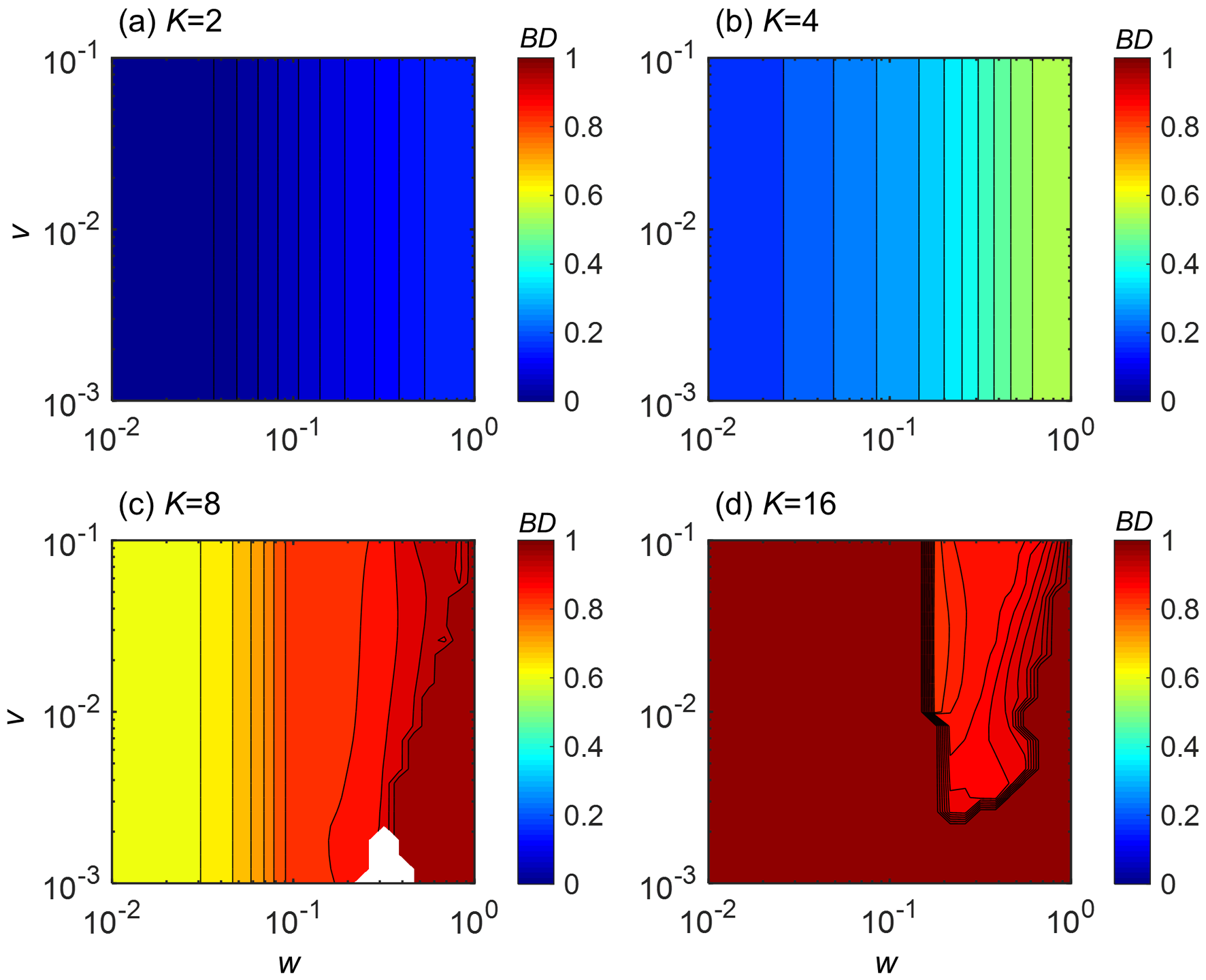


**Figure S8** Dominance of the median biomass (*BD=P/P+C*, across the last 20,000 time steps) of the IG predator, P, over the IG prey, C, in the adaptive intraguild predation module in the parameter space defined by the width *w* and the speed *v* of the trait adaptation with a carrying capacity (*K*) of (a) *K* = 2, (b) *K* = 4, (c) *K* = 8, and (d) *K* = 16. Interpretation of the color-coded values of *BD*: *BD* = 0, P is extinct; 0 < *BD* < 0.5, C dominates; 0.5 < *BD* < 1, P dominates; *BD* = 1, C is extinct. In the white region, C and P are both extinct.


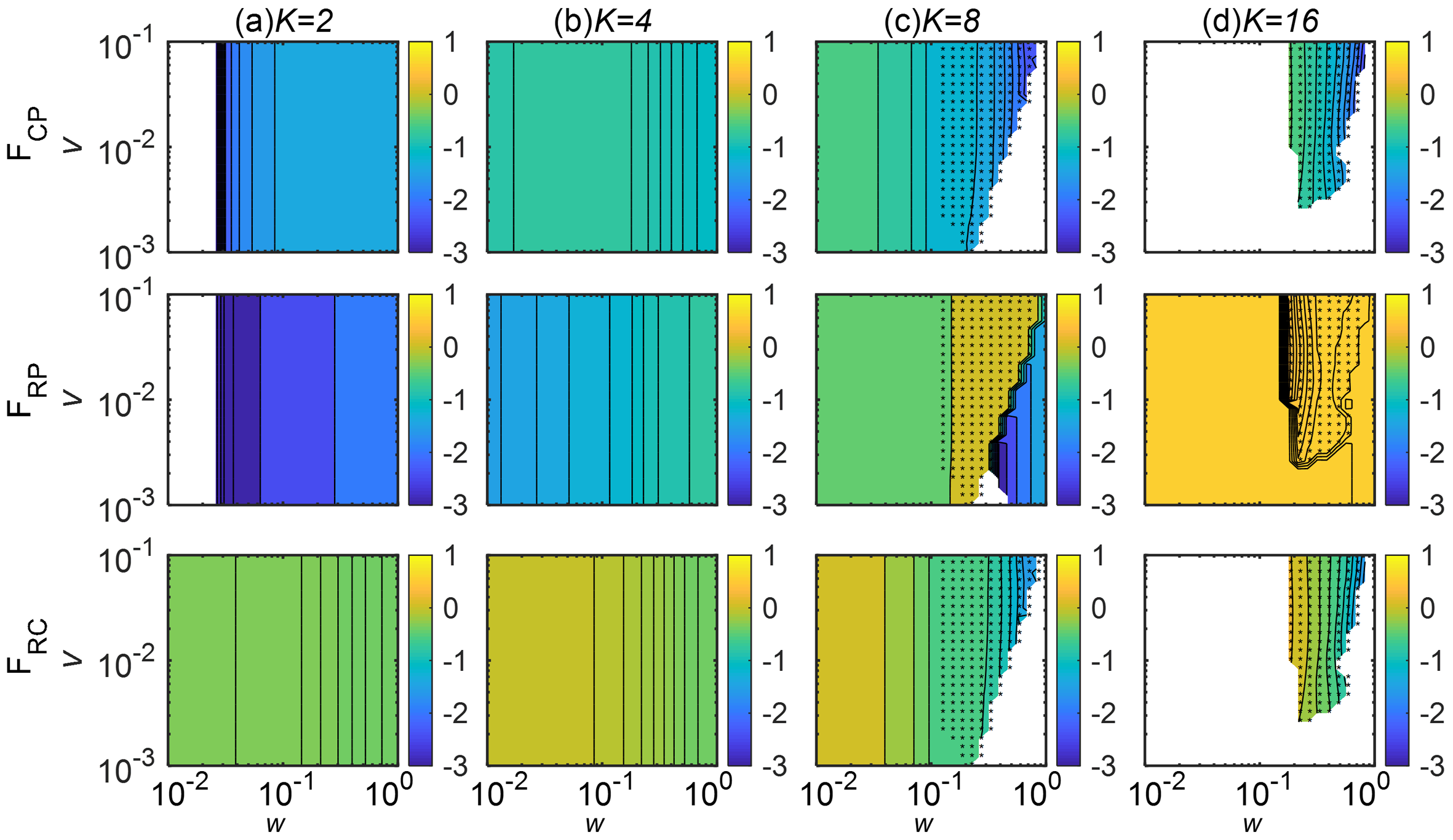


**Figure S9** log_10_(Fluxes) from the IG prey to the IG predator F_CP_ (first row), from the basal prey to the IG predator F_RP_ (second row), and from the basal prey to the IG prey F_RC_ (third row) in the adaptive intraguild predation module in the parameter space defined by the width *w* and the speed *v* of the trait adaptation with a carrying capacity (*K*) of (a) *K* = 2, (b) *K* = 4, (c) *K* = 8, and (d) *K* = 16. White regions indicate the exclusion of either the prey or the predator related to the flux. Regions with and without stars represent oscillatory or steady states, respectively.


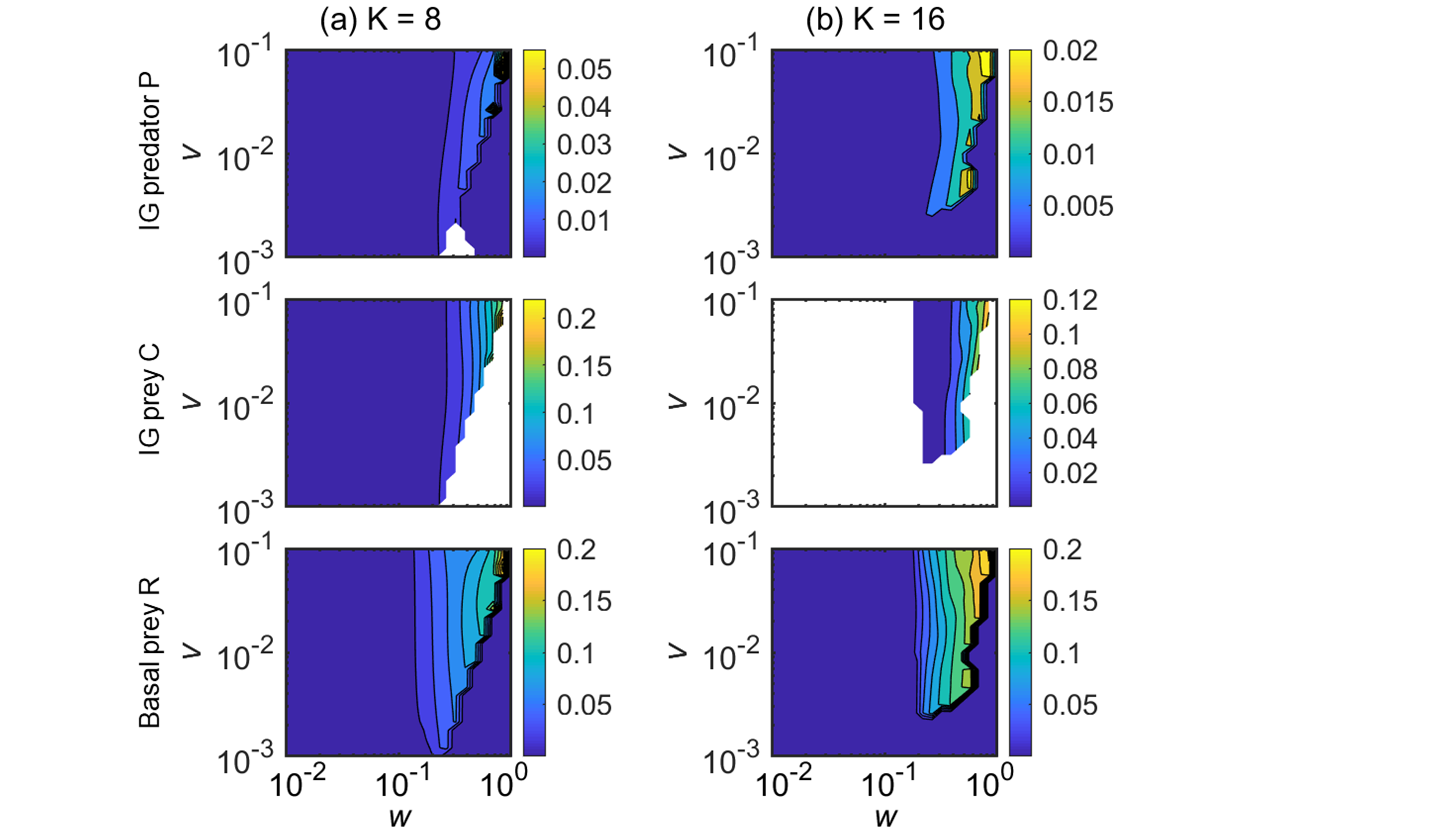


**Figure S10** Standard deviation of trait values across the last 20,000 time steps of the basal prey R, the IG prey C, and the IG predator P in the adaptive intraguild predation module in the parameter space defined by the width *w* and the speed *v* of the trait adaptation with a carrying capacity (*K*) of (a) *K* = 8 and (b) *K* = 16. White regions indicate that the species of the respective panel is excluded from the system.


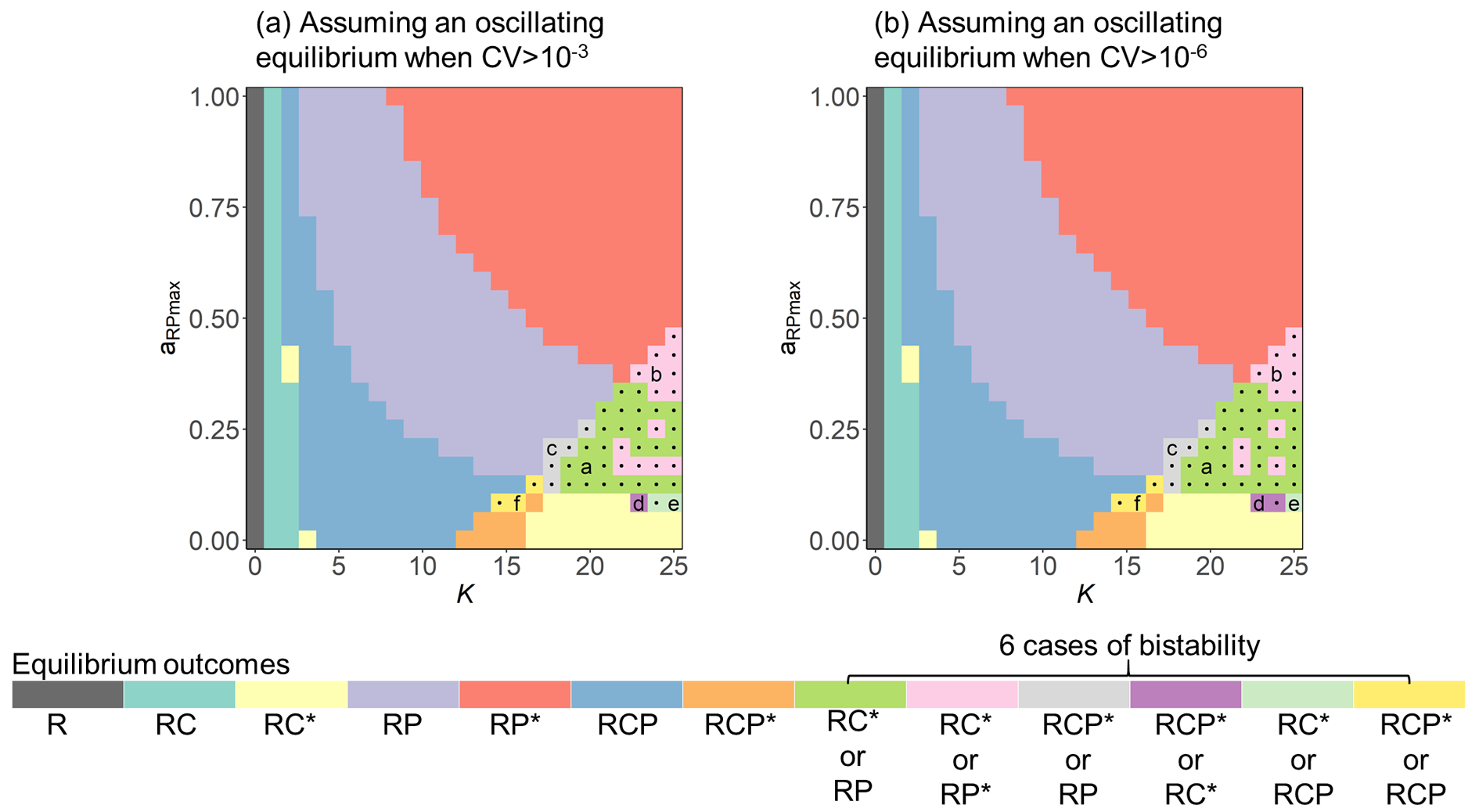


**Figure S11** Equilibrium outcomes of the non-adaptive intraguild predation model in a parameter space defined by the carrying capacity (*K*) and the maximum attack rate of the IG predator on the basal prey (*a_RPmax_*). Oscillating equilibria were assumed when the maximum coefficient of variation (CV) of the biomasses of persisting species was higher than (a) 10^-3^ or (b) 10^-6^. Other parameter values are given in Table 1. Dots mark the region of bistability. a-f represent the 6 parameter combinations between *K* and *a_RPmax_* resulting in the 6 cases of bistability used in Figures S13-S15.


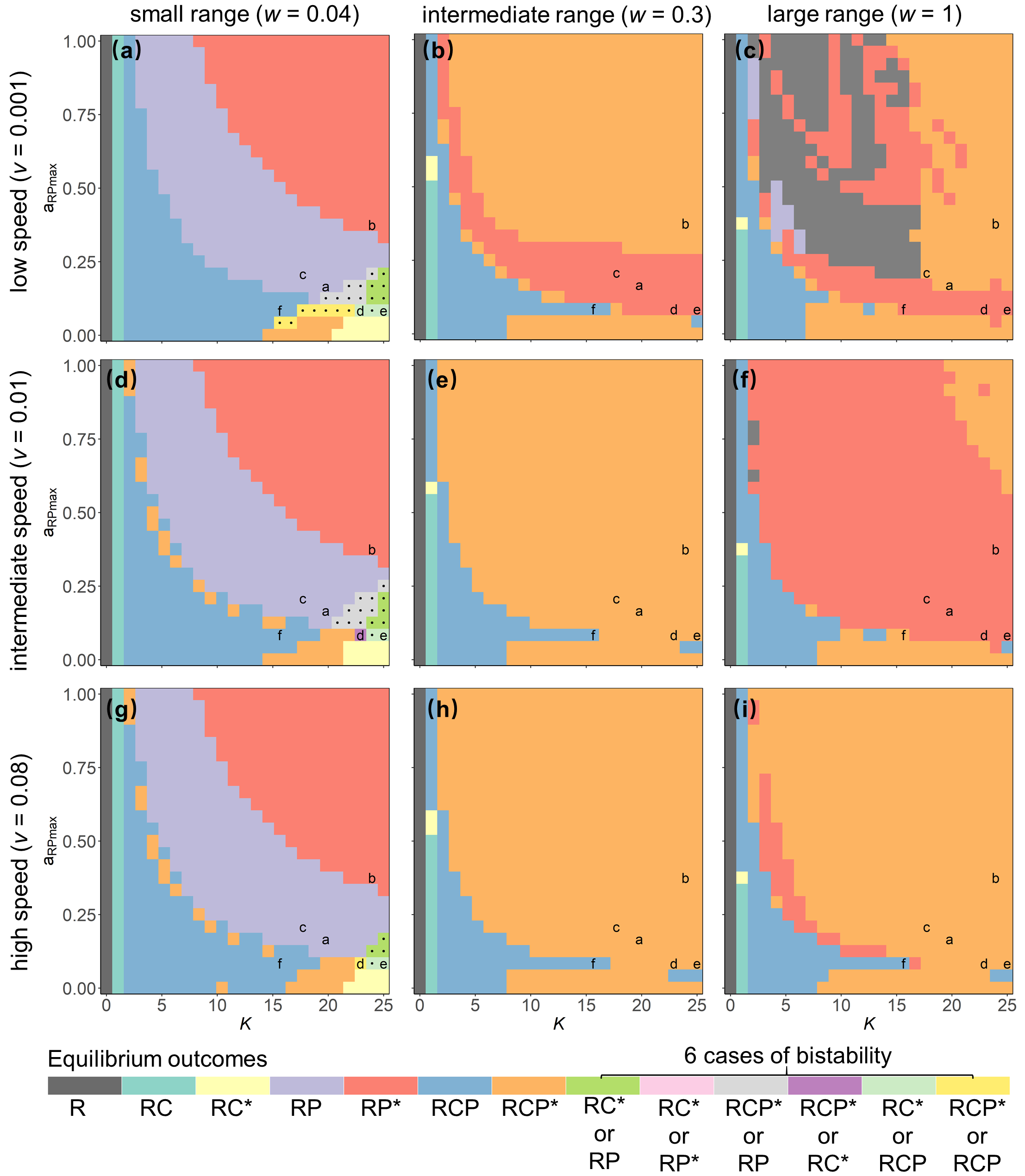


**Figure S12** Equilibrium outcomes of adaptive intraguild predation models in a parameter space defined by the carrying capacity (*K*) and the maximum attack rate of the IG predator on the basal prey (*a_RPmax_*) for different levels of width *w* and speed *v* of trait adaptation. Other parameter values are given in Table 1. Dots mark the region of bistability. a-f represent the 6 parameter combinations between *K* and *a_RPmax_*, resulting in the 6 cases of bistability used in Figures S13-S15. Abbreviations of equilibrium outcomes: R, only R persists; RC, R and C coexist; RP, R and P coexist; RCP, coexistence of the three species. Equilibria marked with * show oscillations. All 6 cases of bistability occurred in the non-adaptive intraguild predation model (cf. Figure 6a in the main text). They were partly also encountered in adaptive models with a small trait width (*w* = 0.04) but not otherwise.


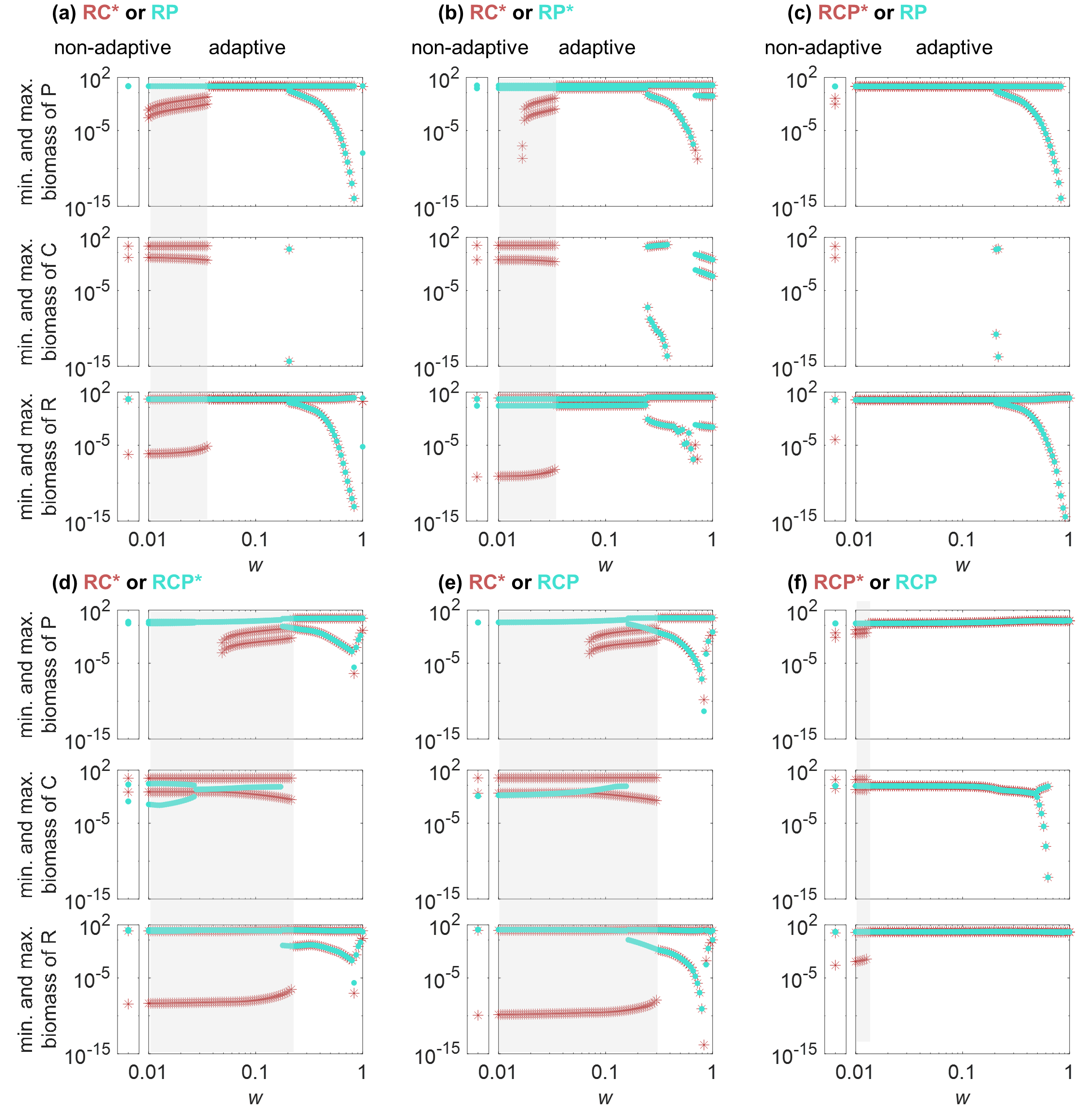


**Figure S13** Bifurcation diagrams showing the minimum and maximum biomasses of the basal prey R, the IG prey C, and the IG predator P in the non-adaptive (left panel) and adaptive (right panel) intraguild predation (IGP) models across an increasing width *w* of trait adaptation for a low speed *v* of trait adaptation (*v* = 0.001). Shaded areas indicate the bistability occurring in the adaptive IGP model. (a-f) Each subplot shows one of the 6 cases of bistability presented in the non-adaptive IGP model (Figure 6a). Abbreviations of equilibrium outcomes: R, only R persists; RC, R and C coexist; RP, R and P coexist; RCP, coexistence of all three species. Equilibria marked * show oscillations. Parameters of the carrying capacity *K* and the maximum attack rate of P on R *a_RPmax_* used in this figure were: (a) *K* = 19.79, *a_RPmax_* = 0.17; (b) *K* = 23.96, *a_RPmax_* = 0.38; (c) *K* = 17.71, *a_RPmax_* = 0.21; (d) *K* = 22.92, *a_RPmax_* = 0.08; (e) *K* = 25.00, *a_RPmax_* = 0.08; (f) *K* = 15.63, *a_RPmax_* = 0.08.


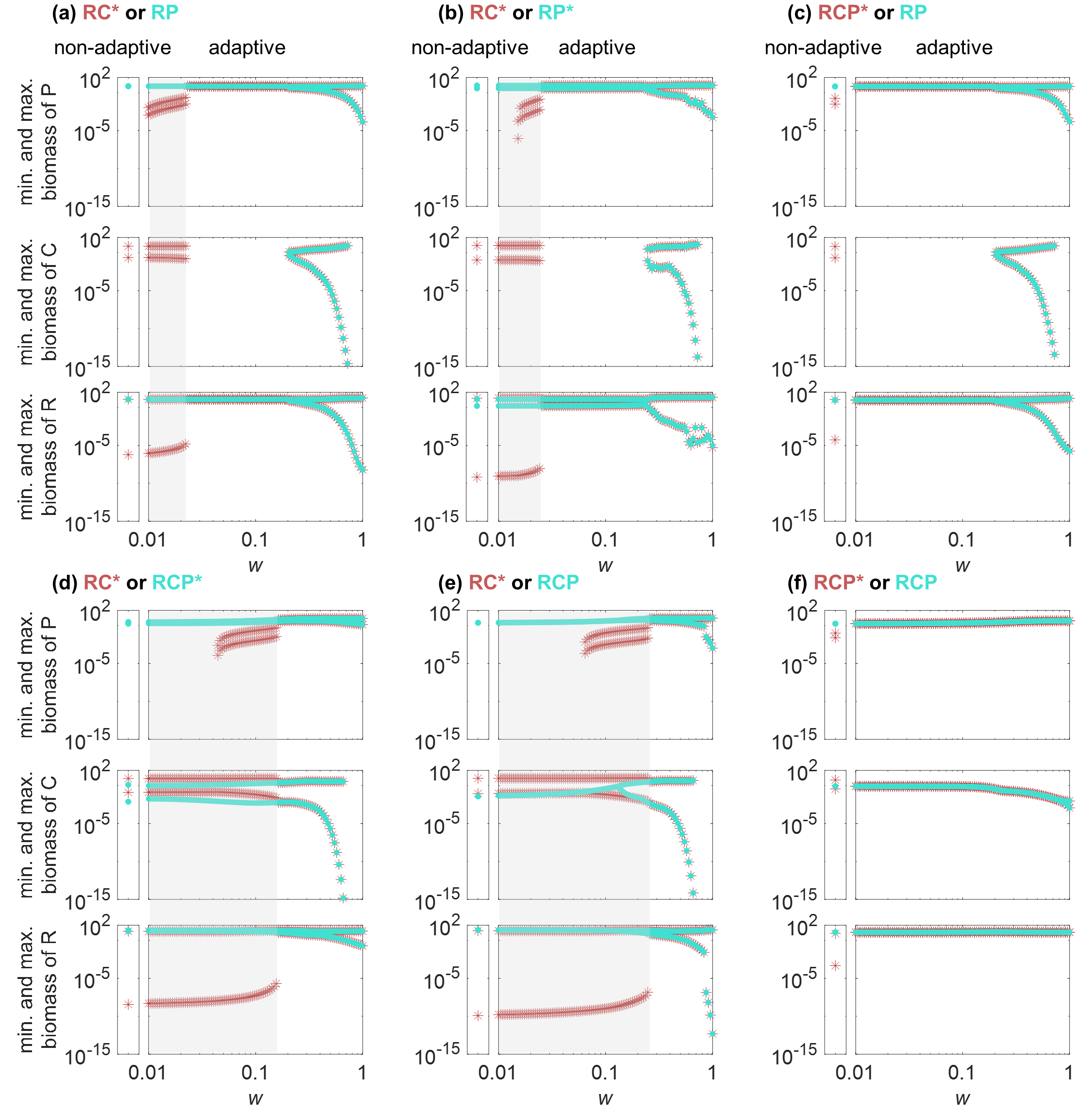


**Figure S14** Bifurcation diagrams showing the minimum and maximum biomasses of the basal prey R, the IG prey C, and the IG predator P in the non-adaptive (left panel) and adaptive (right panel) intraguild predation (IGP) models across an increasing width *w* of trait adaptation for an intermediate speed *v* of trait adaptation (*v* = 0.01). Shaded areas indicate the bistability occurring in the adaptive IGP model. (a-f) Each subplot shows one of the 6 cases of bistability presented in the non-adaptive IGP model (Figure 6a). Abbreviations of equilibrium outcomes: R, only R persists; RC, R and C coexist; RP, R and P coexist; RCP, coexistence of all three species. Equilibria marked * show oscillations. Parameters of the carrying capacity *K* and the maximum attack rate of P on R *a_RPmax_* used in this figure were: (a) *K* = 19.79, *a_RPmax_* = 0.17; (b) *K* = 23.96, *a_RPmax_* = 0.38; (c) *K* = 17.71, *a_RPmax_* = 0.21; (d) *K* = 22.92, *a_RPmax_* = 0.08; (e) *K* = 25.00, *a_RPmax_* = 0.08; (f) *K* = 15.63, *a_RPmax_* = 0.08.


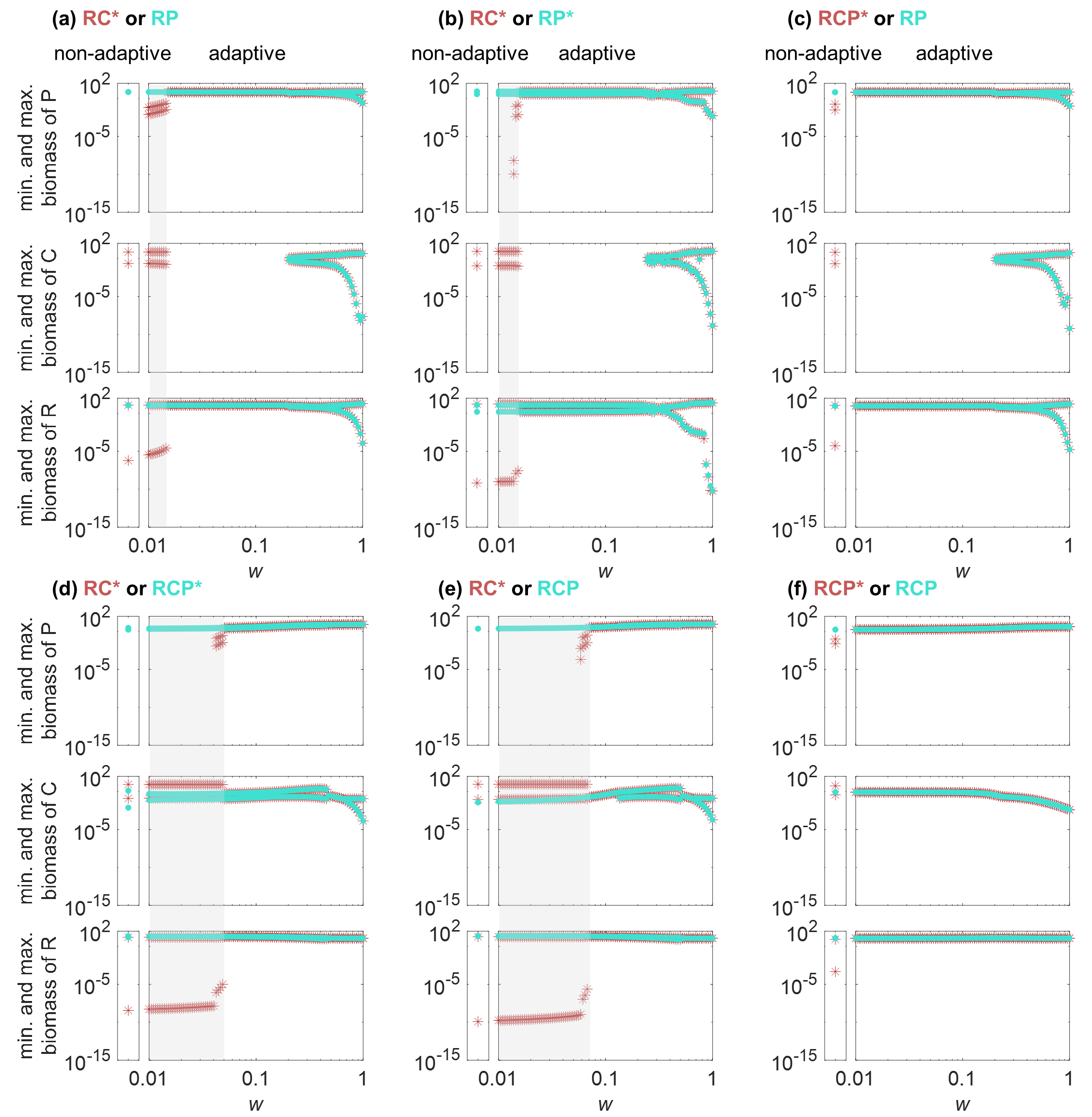


**Figure S15** Bifurcation diagrams showing the minimum and maximum biomasses of the basal prey R, the IG prey C, and the IG predator P in the non-adaptive (left panel) and adaptive (right panel) intraguild predation (IGP) models across an increasing width *w* of trait adaptation for a high speed *v* of trait adaptation (*v* = 0.08). Shaded areas indicate the bistability occurring in the adaptive IGP model. (a-f) Each subplot shows one of the 6 cases of bistability presented in the non-adaptive IGP model (Figure 6a). Abbreviations of equilibrium outcomes: R, only R persists; RC, R and C coexist; RP, R and P coexist; RCP, coexistence of all three species. Equilibria marked * show oscillations. Parameters of the carrying capacity *K* and the maximum attack rate of P on R *a_RPmax_* used in this figure were: (a) *K* = 19.79, *a_RPmax_* = 0.17; (b) *K* = 23.96, *a_RPmax_* = 0.38; (c) *K* = 17.71, *a_RPmax_* = 0.21; (d) *K* = 22.92, *a_RPmax_* = 0.08; (e) *K* = 25.00, *a_RPmax_* = 0.08; (f) *K* = 15.63, *a_RPmax_* = 0.08.


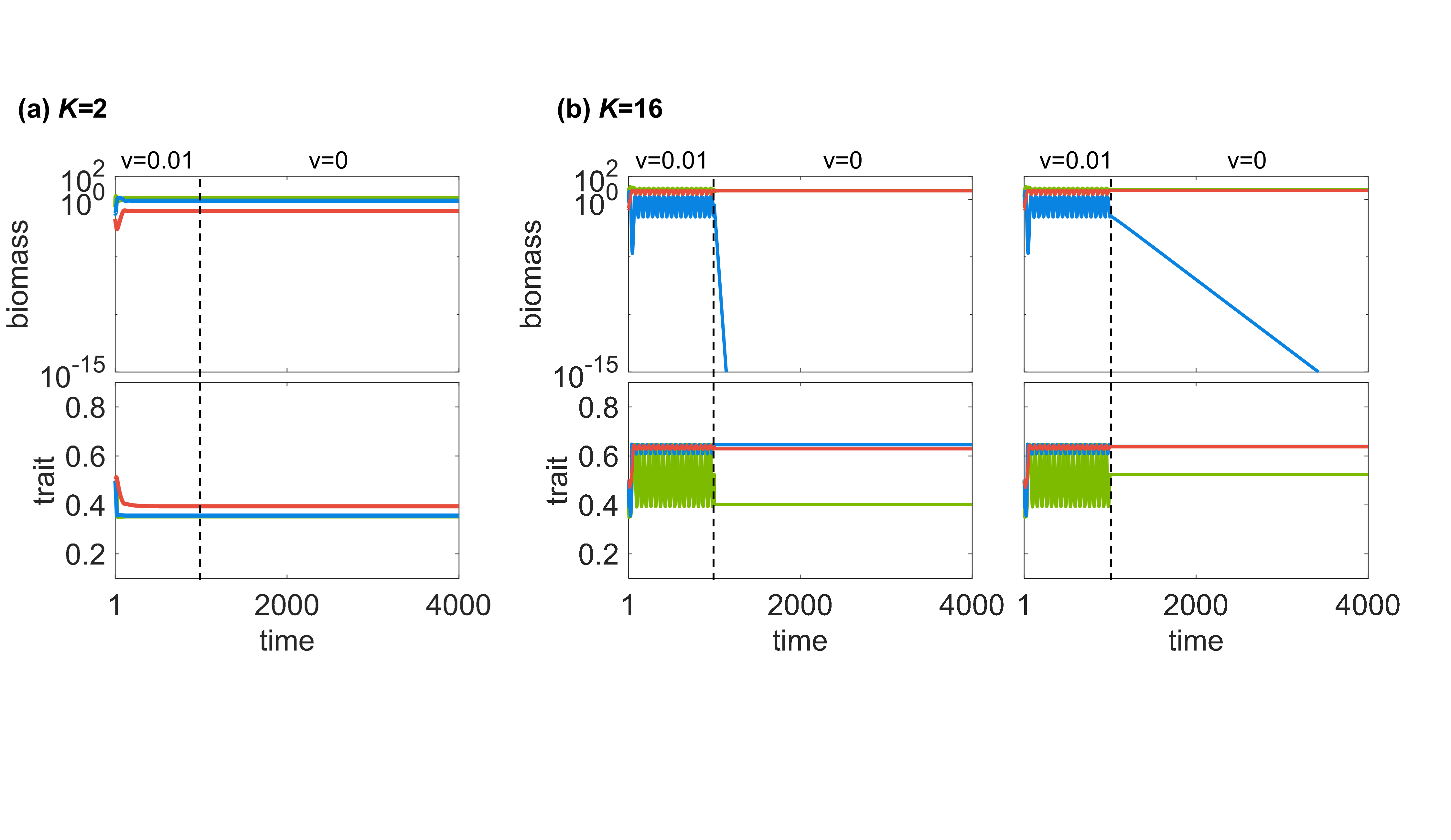


**Figure S16** Biomass and trait dynamics of the basal prey (green line), the IG prey (blue line), and the IG predator (red line) at carrying capacity *K* of (a) 2 and (b) 16. We simulated the adaptive IGP model for the first 1,000 time steps by assuming the speed (*v*) and the width (*w*) of trait adaptation as 0.01 and 0.3, respectively. Then we removed the trait adaptation (*v*=0) and used the final biomass and trait values of the simulated adaptive IGP model as initial values for the simulation of the non-adaptive IGP model for the next 3,000 time steps. To generalize our findings, we simulated the non-adaptive IGP model for two different initial conditions corresponding to current biomass and trait values of the adaptive IGP model at the two different time steps of 990 (left panel) and 1,000 (right panel) in (b).

Our results show that for *K*=2 (equilibrium dynamics) switching off the potential to adapt had no further consequences, revealing that the coexistence of all 3 species in the non-adaptive IGP model was due to the previous trait adaptations. In contrast, for *K*=16 (cyclic dynamics) time-varying trait values driven by ongoing biomass-trait feedbacks (e.g. eco-evolutionary dynamics) were essential for coexistence as the IG prey always went extinct after the potential for ongoing trait adaptation had been removed.

.
